# Design and experimental investigation of the grasping system of an agricultural soft manipulator based on FMDS-YOLOv8

**DOI:** 10.3389/fpls.2025.1683380

**Published:** 2025-10-15

**Authors:** Yu Zhuang, Kunlin Xu, Ziqi Liu, Jiayi Li, Liuyang Shen, Jinfeng Wang

**Affiliations:** College of Engineering, Northeast Agricultural University, Harbin, China

**Keywords:** variable-structure manipulator, grasping system, visual recognition, motion control, FMDS-YOLOv8 model

## Abstract

In response to the need for non-destructive sorting and grasping of fruits and vegetables with diverse sizes and shapes, this study presents a novel design for an agricultural manipulator grasping system (MGS). The system includes a variable-structure soft manipulator equipped with three independently rotatable and distance-adjustable soft actuators. The manipulator can grasp objects with a diameter of ≤140 mm in the center grasping configuration and ≤105 mm in the parallel grasping configuration. An improved FMDS-YOLOv8 vision recognition algorithm was used to detect the type, contour and positional coordinates of the target fruit. A MATLAB-based program was developed to extract the contours of the target fruit and calculate the visualization of the optimal attitude of the soft manipulator. This program facilitated autonomous structural adjustments and precise control during grasping operations. The variable-structure soft MGS was evaluated based on the performance of each component. The experimental results showed a grasping success rate of 95.83%, a grasping damage rate of 4.17%, and a grasping time of about 6.36 s under multi-objective conditions. This verifies the effectiveness and adaptability of the MGS. By adjusting the drive pressure and servo angle, the MGS can grasp fruit and vegetables of different sizes and shapes within its working range, while minimizing damage during the grasping process.

## 1 Introduction

Fruit and vegetables are indispensable components of the human diet. Ensuring their quality and safety not only impacts agricultural economic development and farmers’ income growth (Li et al., 2019), but also serves as a crucial driver for agricultural modernization. The harvesting and sorting of fruit and vegetables heavily rely on manual labor, ranking among the most labor-intensive and time-consuming processes in agricultural production (Ma et al., 2024). Given the intensifying shortage of agricultural labor and the continuous rise in labor costs, the development of efficient and intelligent harvesting equipment has become an urgent necessity (Mana et al., 2024; Treeamnuk et al., 2010).

Machine vision technology, as a key perception method for agricultural robots, has significantly enhanced operational capabilities in complex environments (Zhang and Xu, 2018). However, the growth state, morphological diversity, light variations, and mutual occlusion of fruit and vegetables in natural settings severely impact the recognition accuracy and robustness of visual recognition systems (Tang et al., 2020; Lin et al., 2020). Existing detection algorithms are primarily optimized for single-category fruits. For instance, Zheng et al. (2021) and Xiong et al. (2020) achieved citrus recognition and small object detection based on YOLO BP and Des-YOLO v3, respectively. Nevertheless, these approaches still exhibit insufficient generalization capabilities in scenarios involving the mixed harvesting of heterogeneous, multi-category fruit and vegetables.

As the component directly manipulating fruit and vegetables, the performance of end effectors directly impacts grasping success rates and fruit yield (Yuseung et al., 2024; Gao et al., 2022). The wide variety of produce, coupled with significant morphology and structure differences, as well as their fragile and easily damaged nature, necessitates effectors that are compliant, adaptive, and precisely controllable effectors (Chang and Huang, 2024). Although traditional rigid manipulators can perform grasping tasks, they often cause mechanical damage. For instance, the tomato-picking robot developed by Tang (2018) had a grasping success rate of only 76.3%, highlighting the limitations of rigid structures when handling living objects.

**Figure 1:**
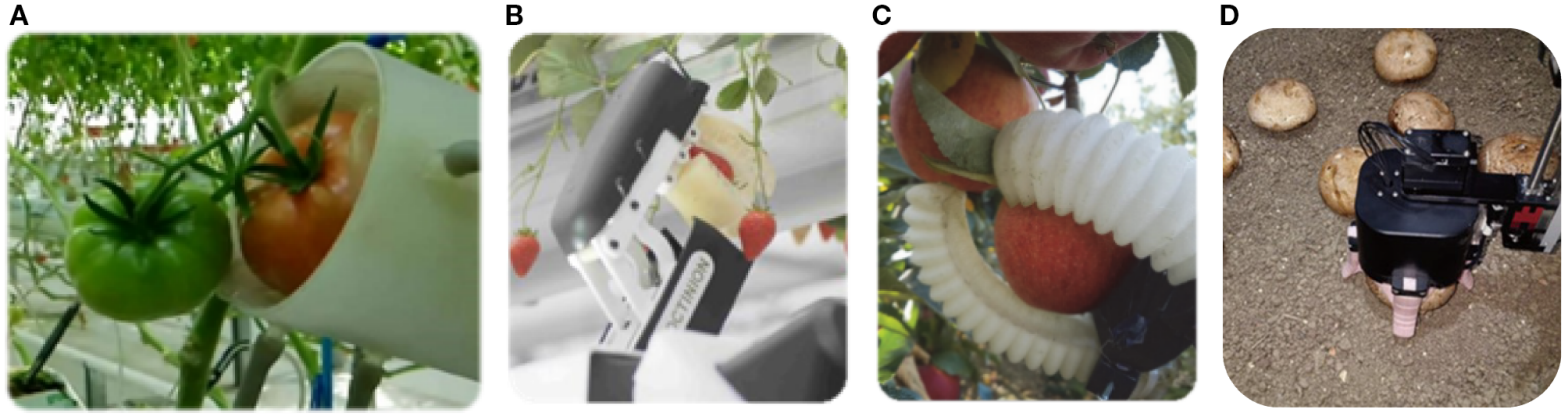
**(A)** Tomato picking end effector; **(B)** Strawberry picker claw; **(C)** Pneumatic end effector; **(D)** Underactuated soft gripper.

The emergence of soft robotics technology offers new avenues for reducing harvest damage (Jones et al., 2021; Choe et al., 2023). Feng et al. (2018) designed a pneumatic end-effector (**Figure 1A**) that utilizes negative pressure suction and pneumatic envelopment to harvest tomatoes. While this achieves low damage rates, it exhibits poor grasping stability. Its adaptability to non-spherical targets (e.g., cucumbers) is severely limited, with a success rate of only 65%, indicating significant constraints in its configuration and actuation method. The strawberry-picking actuator (**Figure 1B**) developed by Octinion (Hemming et al., 2016), employs silicone material to enhance conformability. However, its fixed structure and inability to adjust grasping modes make it difficult to adapt to fruit and vegetables of varying sizes and shapes (Jin and Han, 2024). The 3D-printed pneumatic soft gripper (**Figure 1C**) proposed by Hohimer et al. (2019) adapts well to the curved surface of apples, but it cannot handle clustered or elongated objects effectively due to its limited degrees of freedom. Shi et al. (2025) designed a cable-driven underactuated soft gripper (**Figure 1D**) that can measure the diameter of mushroom in real time during grasping. Through position control alone, the gripper achieved non-destructive mushroom harvesting, supported grading operations and averaged 7.5 s per mushroom.

These studies indicate that soft actuators currently suffer from common issues such as limited functionality, low environmental robustness, and poor control precision. During actual harvesting operations, for example, the diverse morphologies of different crops and the complex, dynamic scenarios demand that actuators possess not only compliant and safe characteristics, but also multimodal grasping capabilities and excellent shape adaptability.

In response to the aforementioned research, this study proposes an intelligent MGS that integrates variable-structure soft actuators with multimodal visual perception. the system dynamically adjusts its grasping configuration based on target morphology by designing a pneumatic variable-structure soft end-effector, enabling multi-mode operations ranging from enveloping to pinching. This significantly enhances adaptability and grasping stability for diverse fruit and vegetables. Subsequently, a multimodal vision system based on an enhanced YOLOv8 was developed. By incorporating attention mechanisms and multi-scale feature fusion strategies, it effectively enhances detection capabilities for occluded, low-light, and small-sized fruits. Finally, a vision-grasping collaborative control framework was established, enabling adaptive matching of fruit/vegetable type recognition, localization, and grasping parameters. This provides a comprehensive solution for the unmanned harvesting of diverse crops. Through structural innovation and algorithmic optimization, this study systematically enhances the picking robot’s adaptability to complex produce objects and operational environments. This has significant implications for reducing post-harvest damage and advancing the universalization and intelligence of agricultural robotics.

## 2 Materials and methods

### 2.1 Overall design of the grasping system

**Figure 2:**
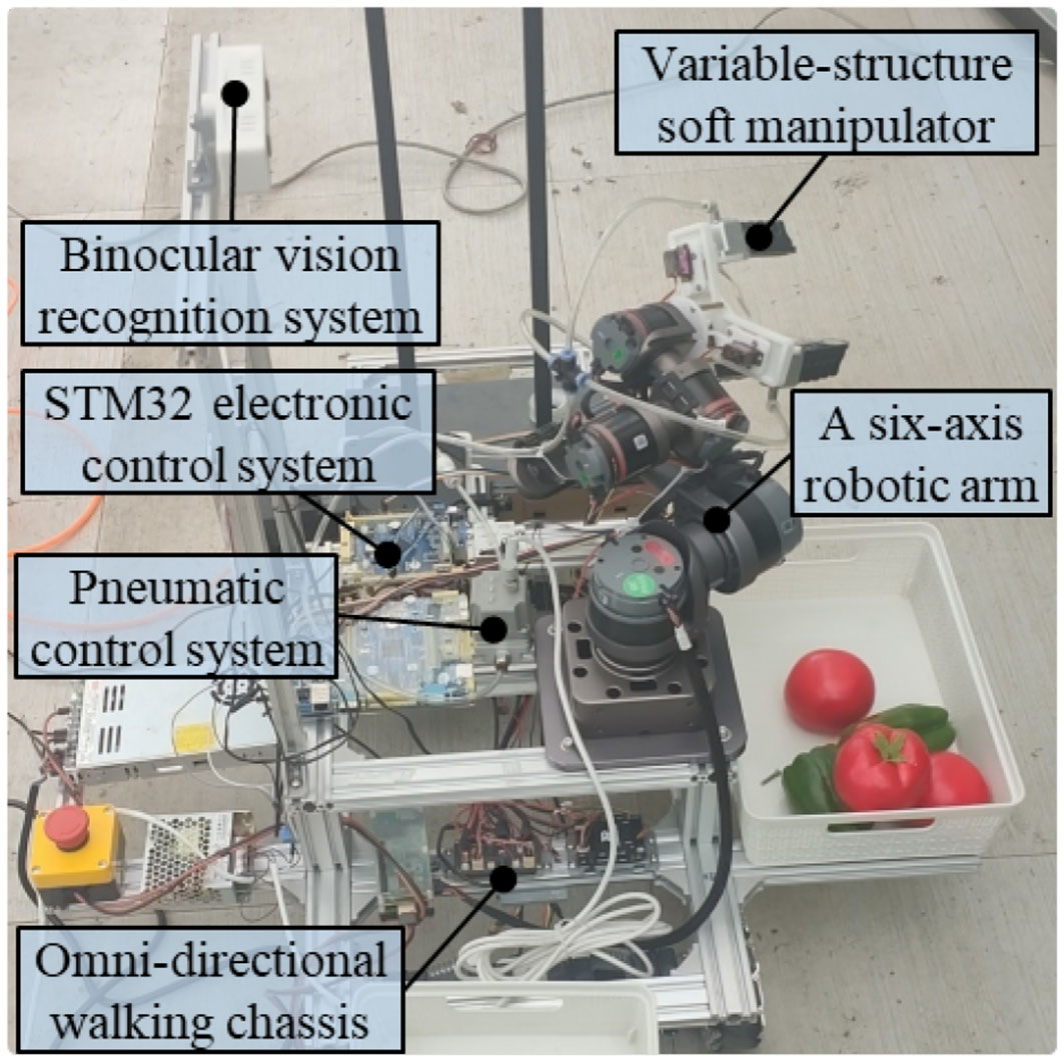
Soft manipulator grasping system.

The soft MGS is mainly composed of pneumatic control system, electronic control system, six-axis robotic arm and variable-structure manipulator (**Figure 2**). Specifically, the pneumatic control system is connected to the manipulator through air hoses and air fittings, including air pumps, pressure regulators and solenoid valves. The electronic control system is mainly composed of ROS host, STM32 microcontroller and DAC module.

Software development plays a key role in realizing the targeted functionality. The software in this study is written in C++ and Python, utilizing ROS Melodic on Ubuntu 18.04. Most of the programs are deployed on the platform’s main computer, which is equipped with a micro-server featuring an AMD Ryzen TM 7 4800H processor with 8 cores and 16 threads. To improve performance, functions such as target detection are processed on the main computer’s graphics processor.

**Figure 3:**
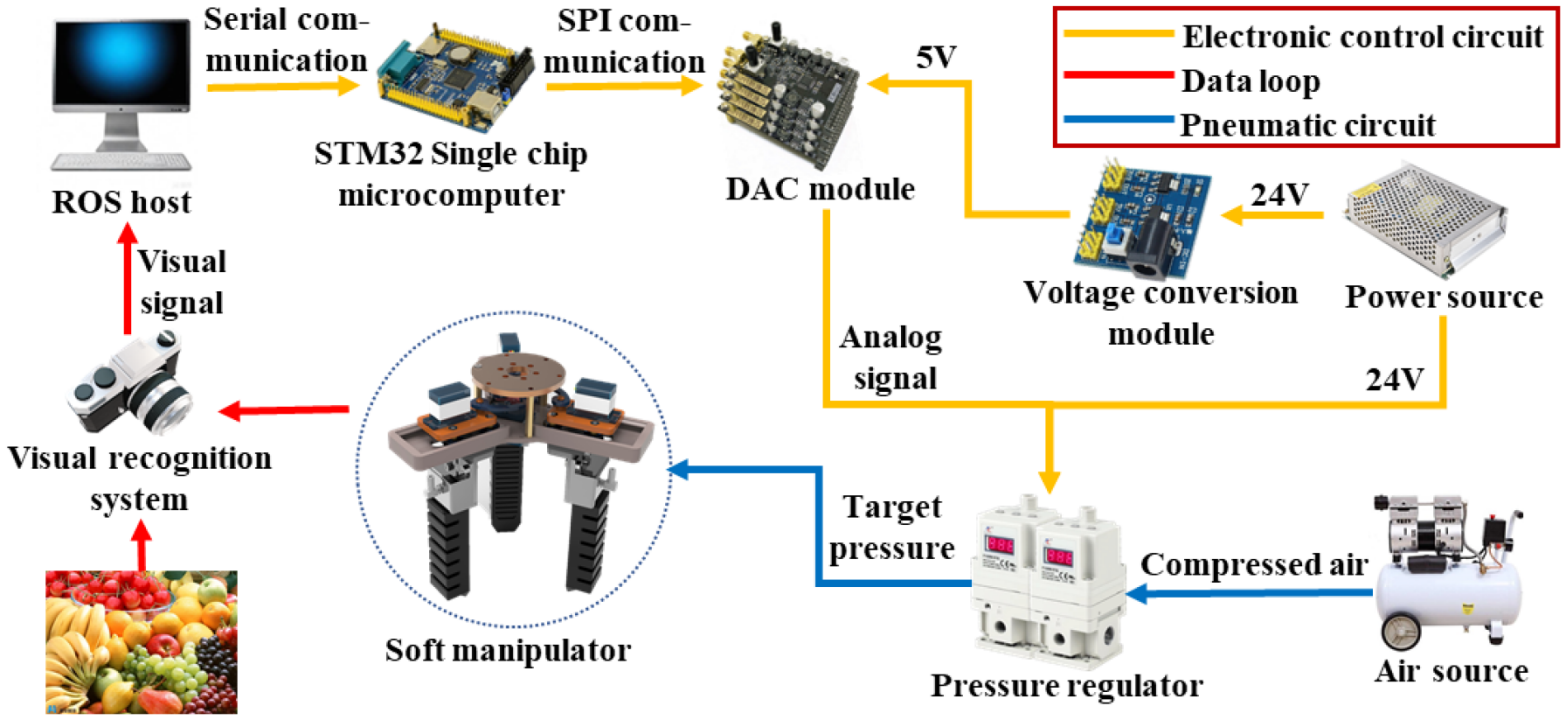
Schematic diagram of the soft manipulator control system.

The schematic diagram of the soft manipulator control system is shown in **Figure 3**. First, the visual recognition system captures the visual signals and transmits them to the host computer for image processing. Subsequently, the ROS host computer coordinates the operation of the six-axis robotic arm to drive the soft manipulator. After reaching the specified target position, the grasping action starts. the STM32 microcontroller sends drive signals to the servo, thus controlling the structural changes of the soft manipulator. Meanwhile, the air source is provided by an air pump, and the input pressure is regulated by a solenoid valve and a precision control valve, both of which are controlled by the microcontroller in order to achieve controllable adjustment of the bending angle under different pressure conditions. By integrating the electrical and pneumatic circuits, the grasping function of the manipulator is fully realized.

#### 2.1.1 Workflow of picking operations

**Figure 4:**
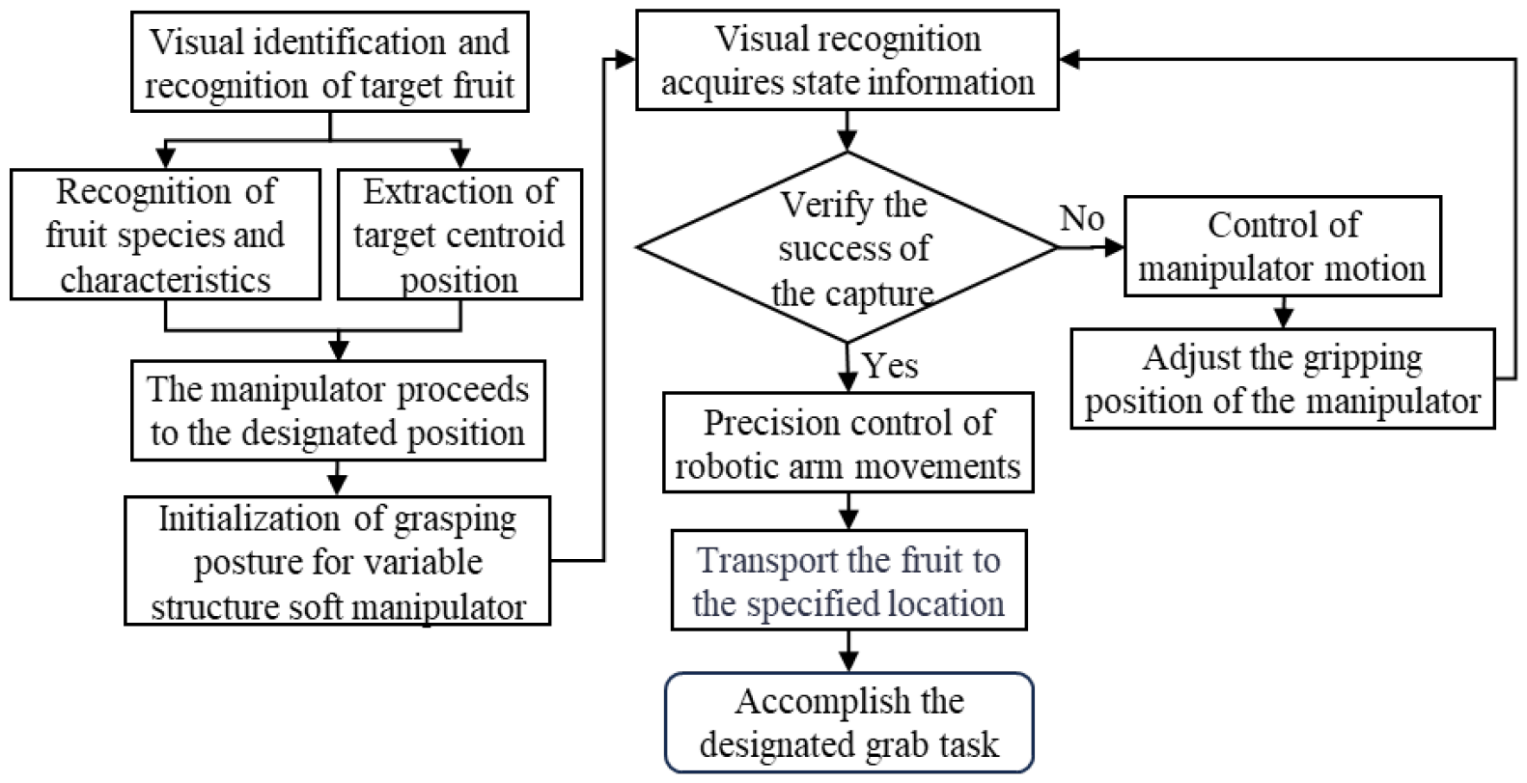
Flowchart for adaptive adjustment control of manipulator grasping posture.

For the detection method of fruit and vegetables, this study used a visual recognition system to detect the key information such as the center and contour of the fruit and vegetables and keep their positions within the predetermined area of the camera image. Based on the visual recognition data, an independently controlled robotic arm and a variable structure soft manipulator complete the grasping of the target products (**Figure 4**). The whole operation process is as follows: firstly, after detecting the target, the agricultural robot transfers the captured image to the main computer to run the developed MATLAB program for image processing, so as to obtain detailed information such as the type, contour and size of the fruit and vegetables. Subsequently, the structure of the manipulator is adjusted according to the detected contours while keeping in line with the position of the manipulator arm in the vicinity of the target. Upon reaching the specified position, the soft actuator is lowered and the drive air pressure is activated, which bends the soft actuator and immobilizes the target. Finally, the manipulator moves to the target position, drops the fruit or vegetable, and then moves to the next target position, repeating the process of recognition, image processing, structure adjustment, and grasping until the picking task is completed.

#### 2.1.2 End-effector structural design

**Figure 5:**
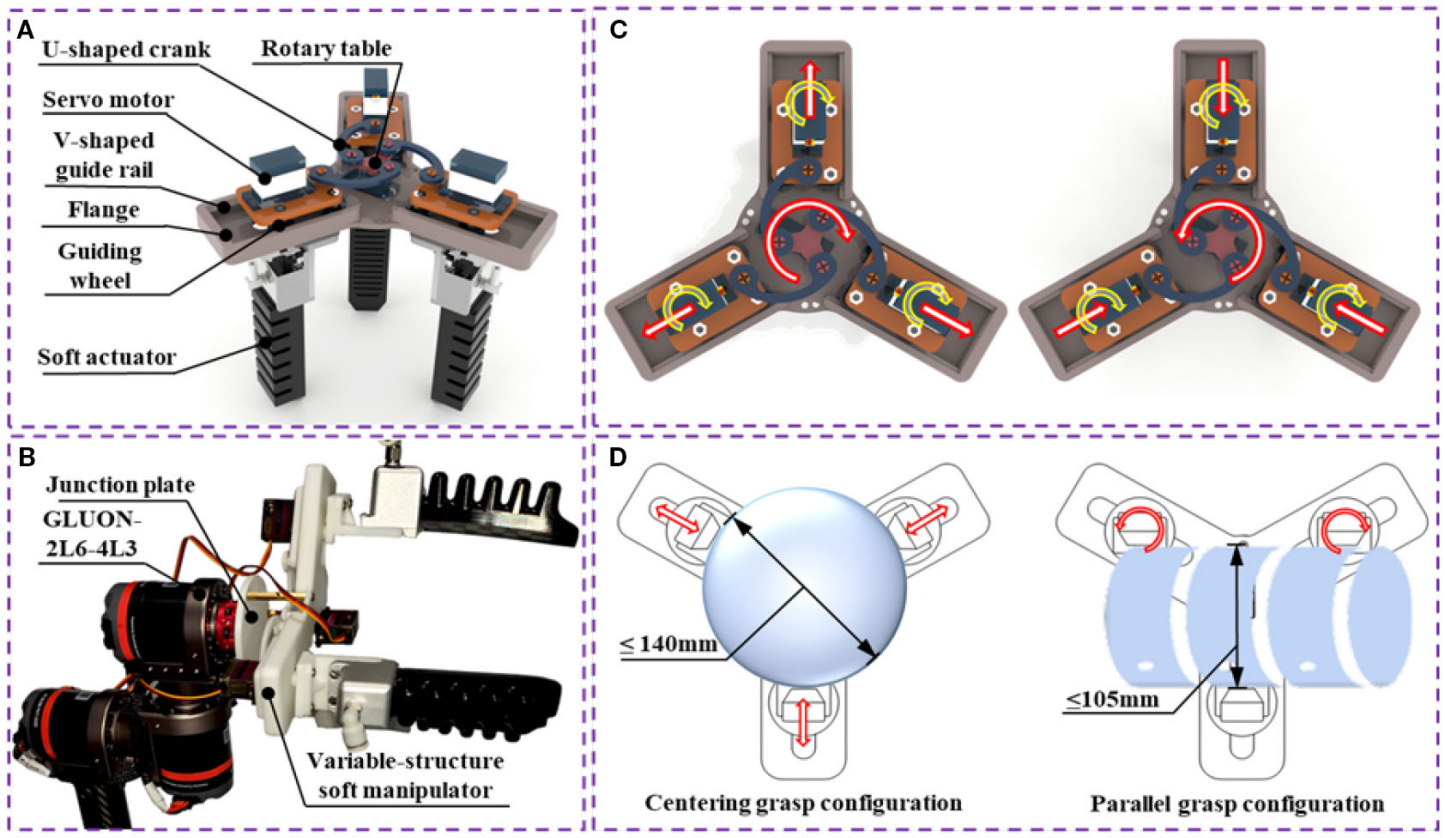
**(A)** Schematic diagram of soft manipulator structure; **(B)** Six-axis mechanical arm architecture; **(C)** Schematic diagram of variable structure motion principle for the soft manipulator; **(D)** The working range of soft manipulator grasping.

In order to adapt to the grasping operation of various fruit and vegetables, a variable structure soft manipulator is proposed in this paper (**Figure 5A**). The manipulator mainly consists of servo motor, flange, rotary table, U-shaped crank, soft actuator and other components. The flange groove is equipped with a V-shaped guideway. The manipulator uses four independent servomotors and three pneumatic soft actuators as the drive mechanism, which is activated by delivering pressurized air through three different pneumatic tubes connected to the pneumatic control system.

The variable-structure soft manipulator is mounted on the GLUON-2L6-4L3 six-axis manipulator via a rotating plate (**Figure 5B**). A central motor, affixed to the substrate, connects to this rotating plate. By controlling the rotation of this central motor, the spacing between the individual software actuators can be adjusted, thereby enabling the expansion and contraction of the entire mechanism. Clockwise rotation of the four components results in the mechanism opening, while counterclockwise rotation causes it to close. The expansion range is defined as *O*_max_ - *O*_min_=3/2×*R_f_*, where *R_f_* represents the flange radius, with a designed range of 36~70 mm. Four independent servo motors are secured to a motor base, which is itself fixed to a slider. This assembly moves along a guide rail together with the slider. The principle and movement of the variable structure are shown in **Figure 5C**.

Because of the complexity and irregularity of the fruit and vegetables to be grasped in reality, we used three independently controlled servo motors mounted on the telescopic mechanism and independently rotated to adapt to the shape of the grasped object, allowing the mechanism to better grasp the direction and position. Three servo motors are connected to the soft actuator, and each soft actuator is controlled separately. The rotation of the servo motor drives the rotation of the three soft actuators, allowing for the variable structure grab of various types of special-shaped fruit and vegetables. As shown in **Figure 5D**, when designing the grasping range of the soft manipulator, this study comprehensively considered the grasping stability of the pneumatic soft actuator and the size range of common fruit and vegetables (such as apples, chillis, oranges, etc., whose large fruit size is generally about 80 mm), so the parallel grasping range was set at 105 mm and the centripetal grasping range was expanded to 140 mm, ensuring applicability within the aforementioned size spectrum.

#### 2.1.3 Manufacture of soft actuators

In this study, we proposed a pneumatic method to enable the single finger actuator to achieve bending motion. Given that fiber-reinforced and multi-cavity fingers are the most prevalent pneumatic soft finger structures, and considering that multi-cavity fingers exhibit a larger bending angle compared to fiber-reinforced fingers, they are better suited for grasping fruit of various shapes and sizes.

**Figure 6:**
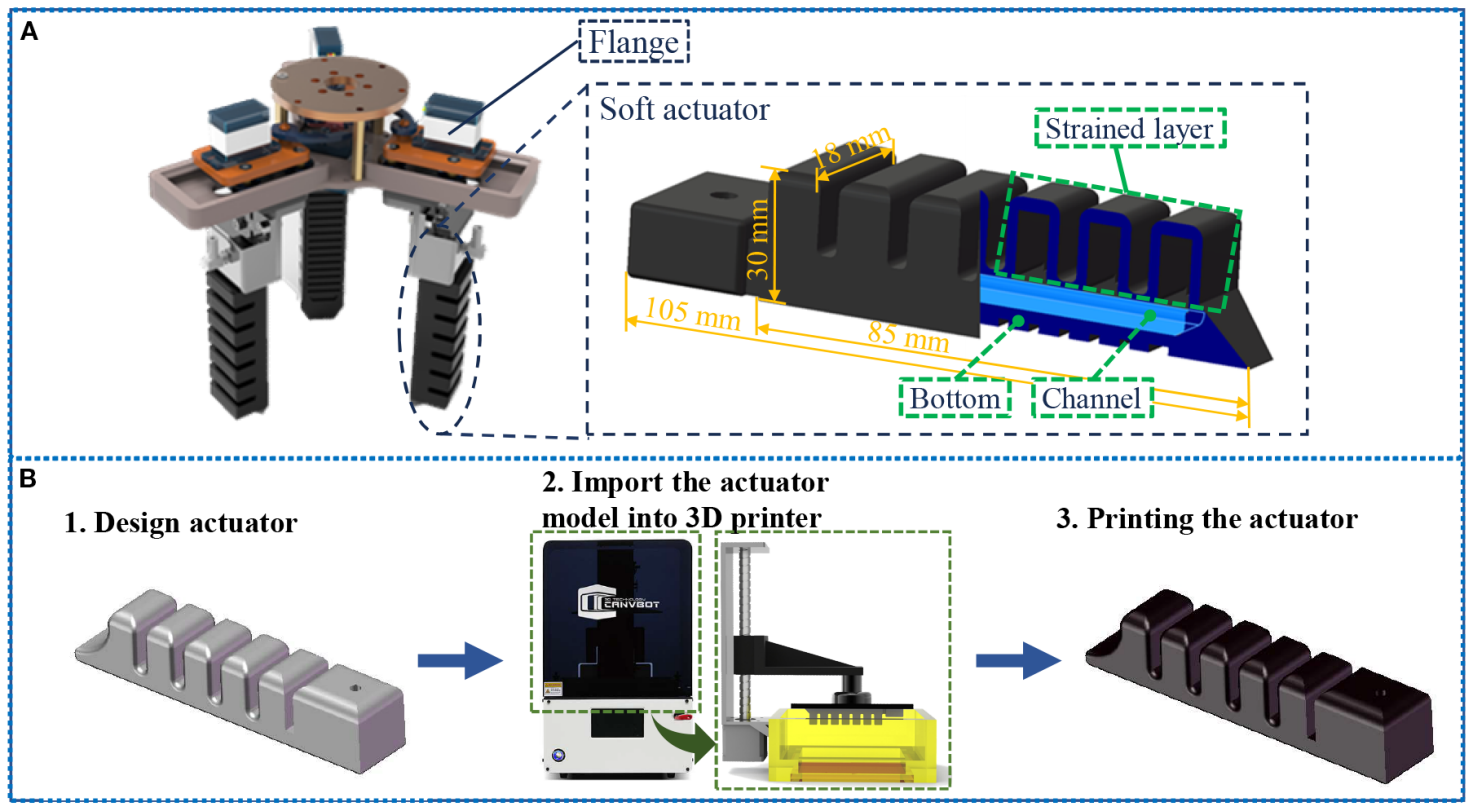
**(A)** The detailed internal architecture of the soft actuator; **(B)** Integrated manufacturing process for the soft actuators.

The detailed internal architecture of the soft actuator is shown in **Figure 6A**. Each soft actuator primarily consists of three components: the strain layer, the channel, and the bottom section. These components are designed with an integrated structure, which allows for significant overall deformation while maintaining relatively low local strain. The overall dimensions of the soft actuator are as follows: 105 mm × 18 mm × 30 mm. Upon an increase in air pressure, the air flows through the channel and fills the chamber, causing the soft actuator to deform and bend. The structural parameters of the soft actuator have been optimized based on our previous research (Zhuang et al., 2023) to minimize energy loss associated with thin-wall radial expansion of the air cavity, thereby limiting radial expansion and enhancing the bending moment.

The rigid components, such as the designed flange and crank telescopic mechanism, are 3D printed using durable PETG material with an Ender-3 S1 Pro printer (Creative 3D Technology Co., LTD., Shenzhen, China). The soft actuator, is fabricated from silicone photopolymer (Resione F80) using a UV 270 light-curing 3D printer (Beijing 3D Robot Technology Co., LTD., Beijing, China). The photocurable resin material was subjected to testing in accordance with the ASTM D412–06 standard, yielding a tensile strength of 7.9 MPa, an elastic modulus of 2.0 MPa, and an elongation at break of 225.1%.

The fabrication process is shown in **Figure 6B**. This includes importing the 3D model of the flexible actuator into Cura software for editing and slicing. The sliced files are then sent to an SLA 3D printer with specific molding parameters set (0.035 mm layer thickness, 8 layers, 30 s bottom layer exposure time, and 10 s exposure time for each layer). F80 silicone resin is poured into the material tray for printing. Post-printing, the soft actuator is cleaned with alcohol and undergoes secondary curing in an oven. Once cured, the solid model of the soft actuator is obtained. Subsequently, the soft actuator is assembled with all mechanical arm parts using bolts, bearings, and other external components to finalize the construction of the variable-structure soft manipulator.

#### 2.1.4 Kinematic modeling of a soft manipulator platform with variable structure

**Figure 7:**
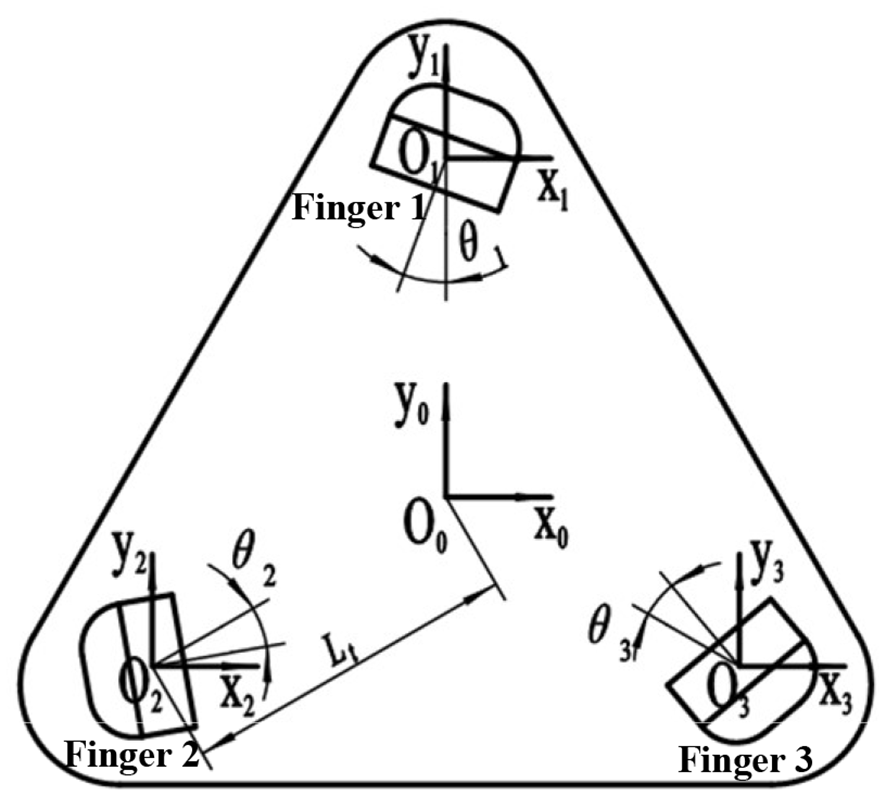
Manipulator and soft actuators coordinate in perpendicular mode.


The center coordinates of the soft manipulator platform and the position coordinates of each actuator root are shown in **Figure 7**. Each kinematic model matrix is represented as follows:

(1) $$A=[\begin{array}{cccc} 0 & L_{0}-R_{f}\cos\theta_{4} & L_{A0} & 0\\ -(L_{0}-R_{f}\cos\theta_{4})\cos\frac{\pi }{6} & -(L_{0}-R_{f}\cos\theta_{4})\sin\frac{\pi }{6} & L_{A0} & 0\\ (L_{0}-R_{f}\cos\theta_{4})\cos\frac{\pi }{6} & -(L_{0}-R_{f}\cos\theta_{4})\sin\frac{\pi }{6} & L_{A0} & 0 \end{array}]$$

(2) $$B=[\begin{array}{cccc} f_{x}(P_{1})\sin\theta_{1} & f_{x}(P_{1})\cos\theta_{1} & f_{z}(P_{1}) & \theta_{1}\\ f_{x}(P_{2})\cos(\frac{\pi }{6}-\theta_{2}) & f_{x}(P_{2})\sin(\frac{\pi }{6}-\theta_{2}) & f_{z}(P_{2}) & \theta_{2}\\ f_{x}(P_{3})\sin(\frac{\pi }{3}-\theta_{3}) & f_{x}(P_{3})\cos(\frac{\pi }{3}-\theta_{3}) & f_{z}(P_{3}) & \theta_{3} \end{array}]$$

(3) $$C=[\begin{array}{cccc} \frac{-\sin\theta_{1}}{abs(\sin\theta_{1})} & \frac{-\cos\theta_{1}}{abs(\cos\theta_{1})} & -1 & 1\\ \frac{\cos(\frac{\pi }{6}-\theta_{2})}{abs[\cos(\frac{\pi }{6}-\theta_{2})]} & \frac{\sin(\frac{\pi }{6}-\theta_{2})}{abs[\sin(\frac{\pi }{6}-\theta_{2})]} & -1 & 1\\ \frac{-\sin(\frac{\pi }{3}-\theta_{3})}{abs[\sin(\frac{\pi }{3}-\theta_{3})]} & \frac{\cos(\frac{\pi }{3}-\theta_{3})}{abs[\cos(\frac{\pi }{3}-\theta_{3})]} & -1 & 1 \end{array}]$$

(4) $$D=[\begin{array}{cccc} x_{1}^{t} & y_{1}^{t} & z_{1}^{t} & \theta_{1}^{t}\\ x_{2}^{t} & y_{2}^{t} & z_{2}^{t} & \theta_{2}^{t}\\ x_{3}^{t} & y_{3}^{t} & z_{3}^{t} & \theta_{3}^{t} \end{array}]=A+B.*C$$

Among these, the symbols for the variables in each kinematic model matrix are detailed in **Table 1**. Given that the size of the target object grasped by the variable-structure soft manipulator designed in this study is contingent upon the flange radius *R_f_* and the bending degree of the software actuator, we have established the flange radius *R_f_* within the range of 36~70 mm and set the effective working length of the soft actuators at 85 mm.

**Table 1:** Nomenclature for kinematic model of variable structure platform.

Symbol	Description
*θ*_1_ ∼ *θ*_3_	Servomotor rotation angle for controlling soft actuator, when the pointer points (O_0_) to the origin, *θ*_1_ ∼ *θ*_3_ = 0°, clockwise is positive and counterclockwise is negative
*θ*_4_	The rotation angle of the middle servo motor, When the variable-structure soft manipulator is in the grasping position, *θ*_4_ = 0°, clockwise is positive and counterclockwise is negative
*P*_1_~*P*_3_	Input air pressure of soft actuator
*L*_0_	The maximum distance between the center of soft actuator and the origin when t the variable structure soft manipulator is fully extended
*R_f_*	Radius of the flange (The soft actuator allows for adjustable distance settings based on the flange center)
*L_A0_*	Initial length of soft actuator
*f_x_* (*P*)	The horizontal displacement of the end of the soft actuator during its bending movement
*f_z_* (*P*)	The vertical displacement of the end of the soft actuator during its bending movement
A	XY plane transformation matrix driven by servomotor 4. As shown in Equation 1
B	XYZ space matrix caused by the soft actuator. As shown in Equation 2
C	Transfer matrix generated by servomotors 1 ~ 3. As shown in Equation 3
D	Spatial location and direction matrix of the soft actuators ends. As shown in Equation 4

### 2.2 Fruit and vegetable testing

#### 2.2.1 Data collection and dataset creation

**Figure 8:**
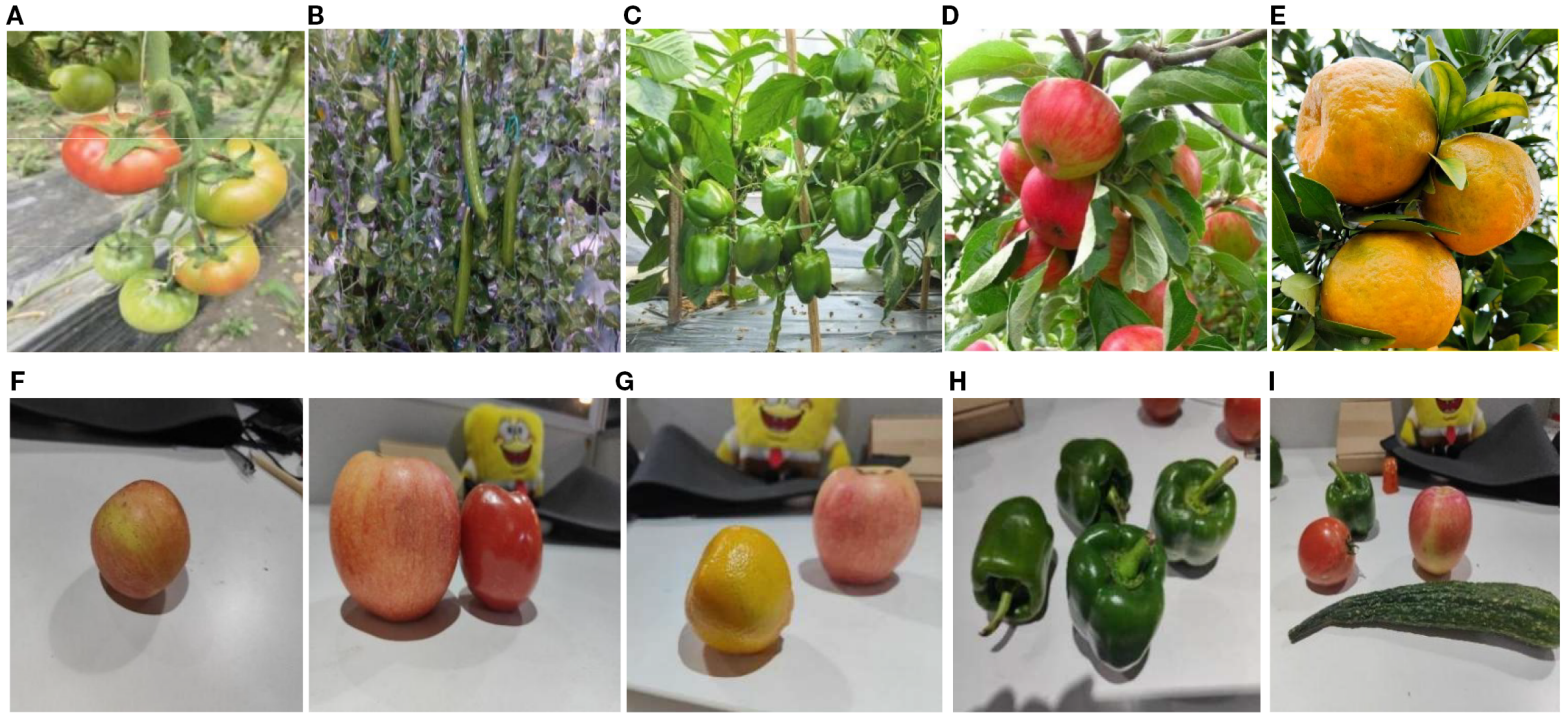
Homemade datasets: **(A)** Tomato; **(B)** Cucumber; **(C)** Chilli; **(D)** Apple; **(E)** Orange; **(F)** Single fruit; **(G)** Various types of fruits with similar characteristics; **(H)** Same species multiple fruits; **(I)** Various types and multiple fruits.

The images used were collected from Xiangyang Farm of Northeast Agricultural University between late May and late September 2024, during the time slots of 10:00–12:00 and 14:00–17:00. To address the “domain gap” issue arising from the transition of agricultural harvesting robots from structured laboratory environments to unstructured field environments, this study strategically incorporated images captured under laboratory conditions during data construction. This strategy aims to provide the model with a progressive learning framework, transitioning from simple, controlled conditions to complex, variable conditions. This enhances its fundamental feature extraction capabilities, laying a solid foundation for eventual real-world field deployment. Images were captured using a smartphone at a resolution of 4016 pixels × 3012 pixels. During capture, images were taken from multiple angles—including overhead, side, and upward views—at distances ranging from 0.3 to 2.5 meters from the fruit, simulating the perspective of field operations. This covered typical lighting scenarios such as strong midday sunlight on clear days, diffuse light under cloudy conditions, and shadow obstruction. After screening, 400 valid sample images were retained for each category of fruit and vegetable data, totaling 2,000 images. The data information is shown in **Figure 8**.

From the raw dataset, 1,600 images were randomly selected at an 8:1:1 ratio to form the training set, 200 images for the validation set, and 200 images for the test set. All images were uniformly resized to 640 pixels × 640 pixels. Subsequently, labeling was employed to annotate the images, yielding a label matrix. After annotation, the RoboFlow platform implemented multimodal data augmentation strategies including random rotation, perspective transformation, brightness and contrast adjustment, noise injection, and motion blur. This enhanced the model’s robustness to lighting fluctuations and motion blur. Following augmentation, the total dataset expanded to 6,000 images.

#### 2.2.2 Improvements to the YOLOv8 model

Although YOLOv8 has been widely adopted as an advanced detection model, it still faces challenges such as sample overlap, blurred imaging, and target occlusion in detection tasks of fruit and vegetables. The issue of blurred object boundaries under dense distribution conditions significantly reduces detection and localization accuracy, directly impacting the grasping success rate of agricultural harvesting robots.

**Figure 9:**
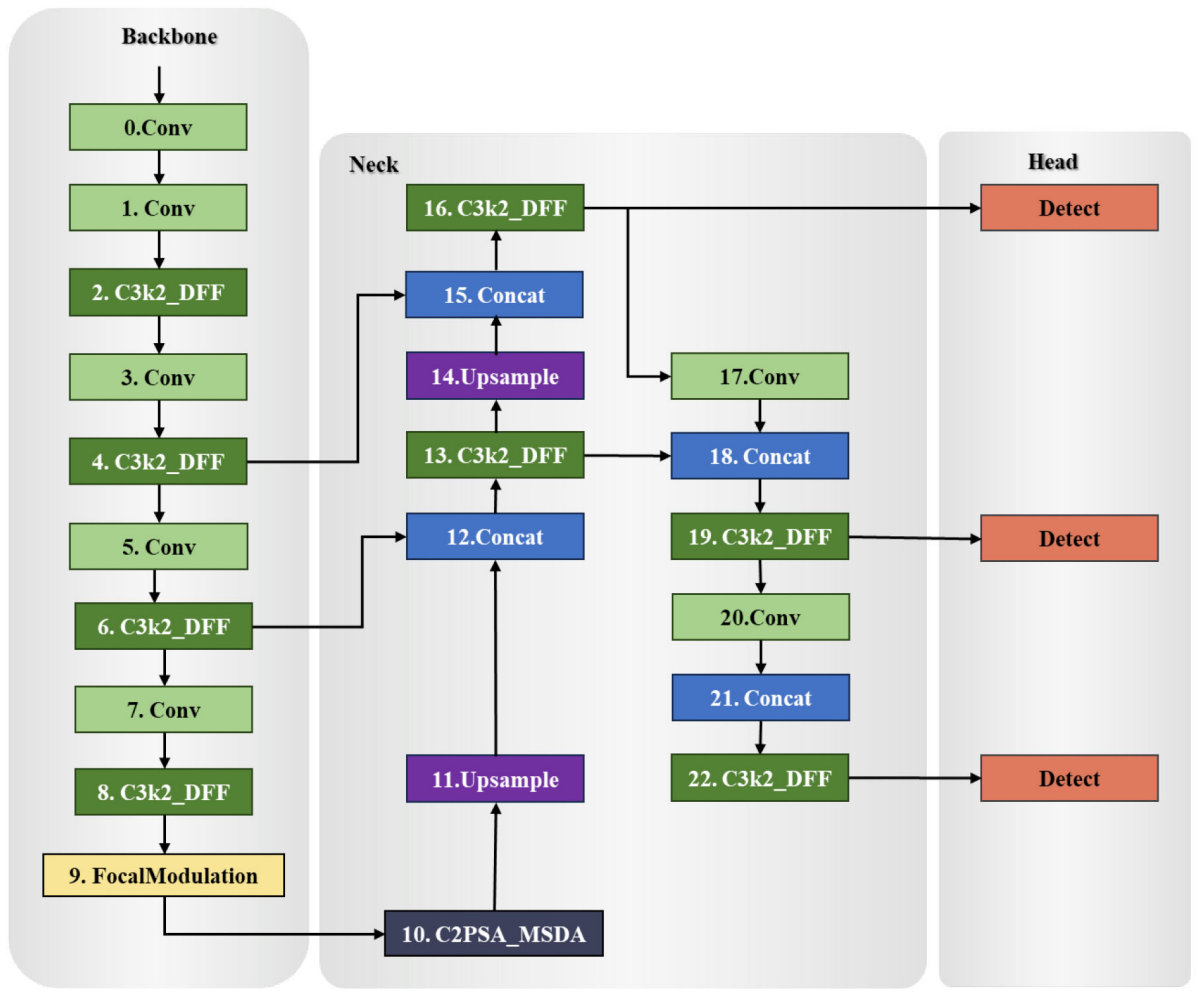
FMDS-YOLOv8 network structure diagram.

To achieve high-precision harvesting, this study proposes a two-stage detection framework based on YOLOv8, named FMDS-YOLOv8 (structure shown in **Figure 9**). This framework first employs an enhanced YOLO model for rapid identification and preliminary localization of fruit and vegetable targets. The detection results are then fed as anchor box proposals into the SAM2 segmentation model to obtain pixel-level segmentation masks and contour information. Finally, by integrating the physical structure and motion constraints of the robotic gripper, the optimal picking point is calculated. By coupling recognition and segmentation modules, this method enhances grasping accuracy and robustness in complex scenarios.

In order to achieve accurate and efficient fruit and vegetable target detection, this study proposes an enhanced fruit and vegetable target detection network FMDS-YOLOv8 based on YOLOv8, as shown in **Figure 9**. The main improvements are as follows:

1. This study proposes an improved model FMDS-YOLOv8 based on YOLOv8, which aims to improve the recognition accuracy and localization speed of targets in grasping scenarios of fruit and vegetables.
2. FMDS-YOLOv8 incorporates three core innovative modules: FocalModulation spatial pyramid pooling, C2PSA_MSDA multiscale feature fusion module, and C3k2_DFF efficient feature extraction structure. Experimental validation shows that the model has significant advantages in recognition accuracy and inference speed.
3. FMDS-YOLOv8 is used for the automation of grasping for fruit and vegetables as well as the intelligent development of agricultural robots, providing a reference for future research in this field.

#### 2.2.3 Feature extraction optimization

##### 2.2.3.1 FocalModulation module

The integration of FocalModulation into the YOLOv8 framework represents a key advancement in enhancing the model’s fruit and vegetable recognition capabilities, especially in complex orchard picking environments. By replacing the original Fast Feature Pyramid Pooling (SPPF) module with FocalModulation, the model significantly improves the processing efficiency and accuracy of fruit and vegetable target recognition and localization, even in complex contexts with branch and leaf occlusion, overlapping fruits, or varying illumination.

**Figure 10:**
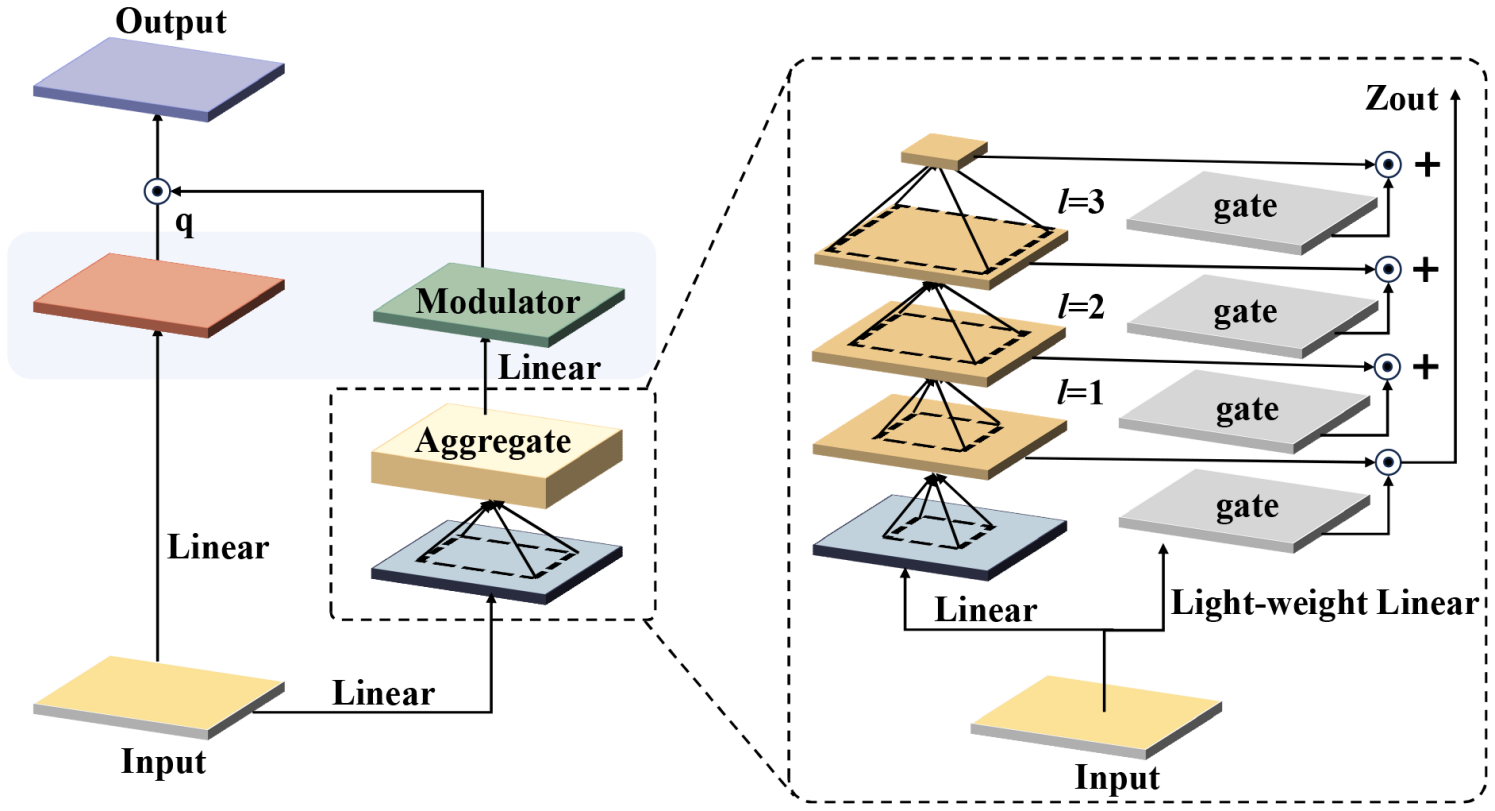
FocalModulation structure.

The core of FocalModulation lies in its dynamic focus adjustment mechanism designed to enhance the model’s perception of key features in the image. This innovative approach allows for meticulous modulation of the model’s focus to direct attention to fruits, specific features, or critical areas, leading to more accurate fruit and vegetable classification, localization, and condition assessment. As shown in **Figure 10**, the structure of FocalModulation demonstrates its unique ability to dynamically adjust the focus of the model, ensuring that the fruit itself and its key attributes are effectively captured and emphasized.

FocalModulation distinguishes itself from the SPPF module by prioritizing the extraction and analysis of core fruit and vegetable features in the image. While SPPF enhances the model’s ability to process multi-scale inputs, FocalModulation goes deeper and improves the model’s focus to increase the accuracy of detection and localization of fruit and vegetable targets. This targeted approach ensures that the diversity of input data is preserved, but the focus is on the elements most critical to accurate fruit and vegetable identification and status determination.

The use of FocalModulation in the YOLOv8 architecture enables the model to exhibit higher discrimination and good performance in fruit and vegetable recognition tasks, especially in scenarios involving densely arranged, occluded, or similar-looking different fruit and vegetable classes. The model’s enhanced adaptability to various input image sizes and its enhanced ability to pinpoint individual fruits or key regions greatly improves its recognition accuracy and robustness.

##### 2.2.3.2 C2PSA_MSDA module

In the YOLOv8 model, the C2PSA module dynamically weights the feature map with Point-wise Spatial Attention (PSA) blocks to enhance feature extraction and representation. This mechanism enables the model to selectively focus on key regions in the image, effectively enhancing the ability to capture subtle key details while suppressing redundant information in the background or interference regions. However, in real-world scenarios of fruit and vegetable recognition, target objects often face complex challenges such as multi-scale variations, branch and leaf occlusion, overlap between fruits, uneven lighting conditions, and confusion of similar appearance categories. These factors impose higher requirements for constructing robust feature representations.

**Figure 11:**
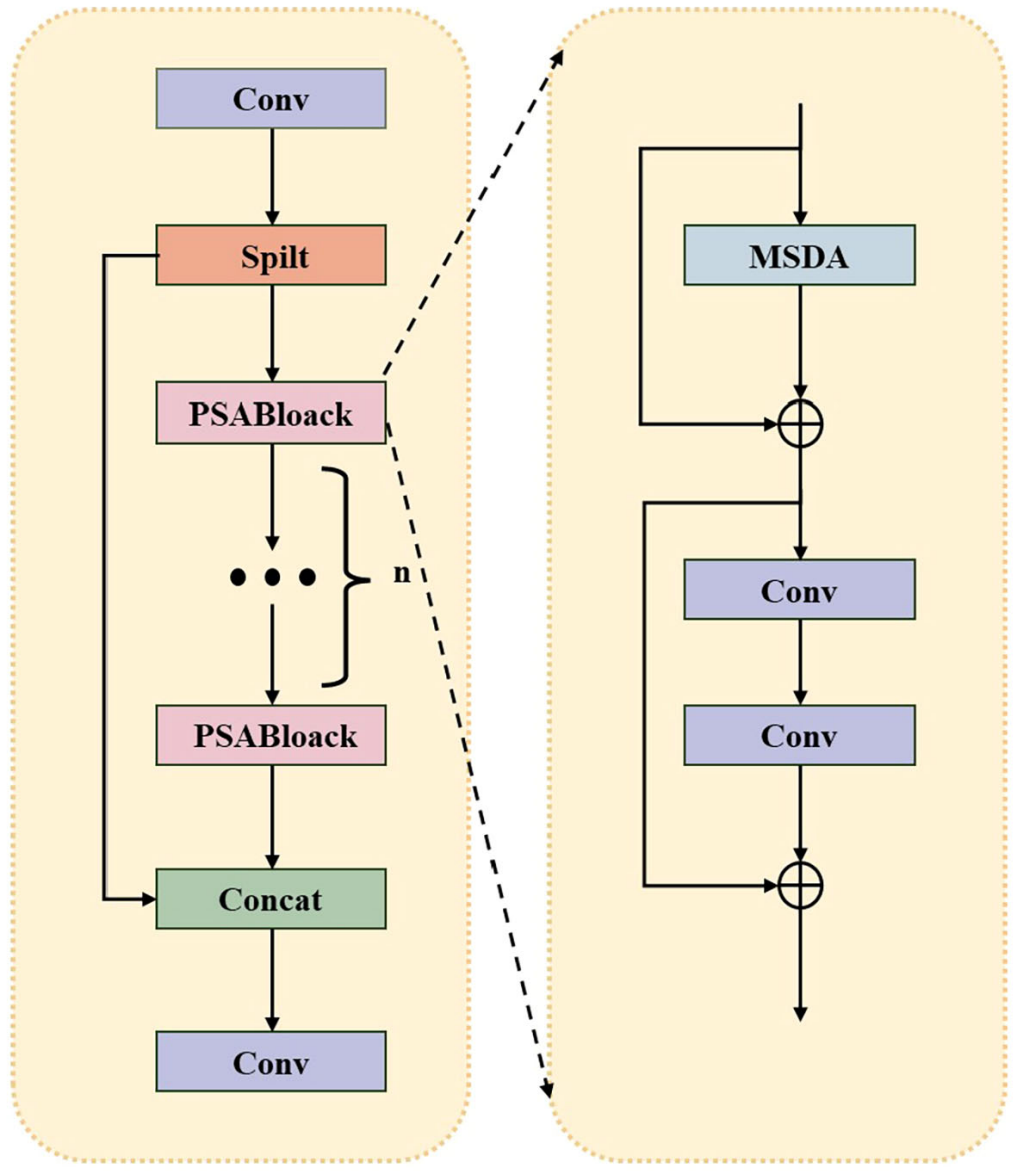
C2PSA_MSDA module structure.

To address these challenges, as shown in **Figure 11**, this study further integrates the Multi-Scale Dilated Attention (MSDA) mechanism into the PSA block to construct the enhanced C2PSA_MSDA module. The core of the MSDA mechanism lies in its dynamic adaptive sensory field adjustment capability, which distinguishes it from the traditional attention mechanism that relies on fixed spatial transformations of traditional attention mechanisms. Enhancing the network’s ability to extract multi-scale contextual information. This adaptive approach enables the model to adaptively adjust the effective receptive field size in the face of drastic changes in target scale, severe partial occlusion, dense overlapping arrangement, uneven lighting conditions or similar category confusion, and accurately retain and utilize key structural details at different scales.

This adaptive fusion of multi-scale contextual information guided by PSA and realized by MSDC greatly enhances the discriminative and robustness of feature representation. It enables the network to localize fruit and vegetable recognition more accurately in complex and changing agricultural environments, and is especially effective in mitigating the negative impacts of image blurring, occlusion and uneven illumination. The parallel sensitivity of the model to fine-grained details and large-scale semantic context synergistically improves the localization accuracy and classification accuracy of detection.

**Figure 12:**
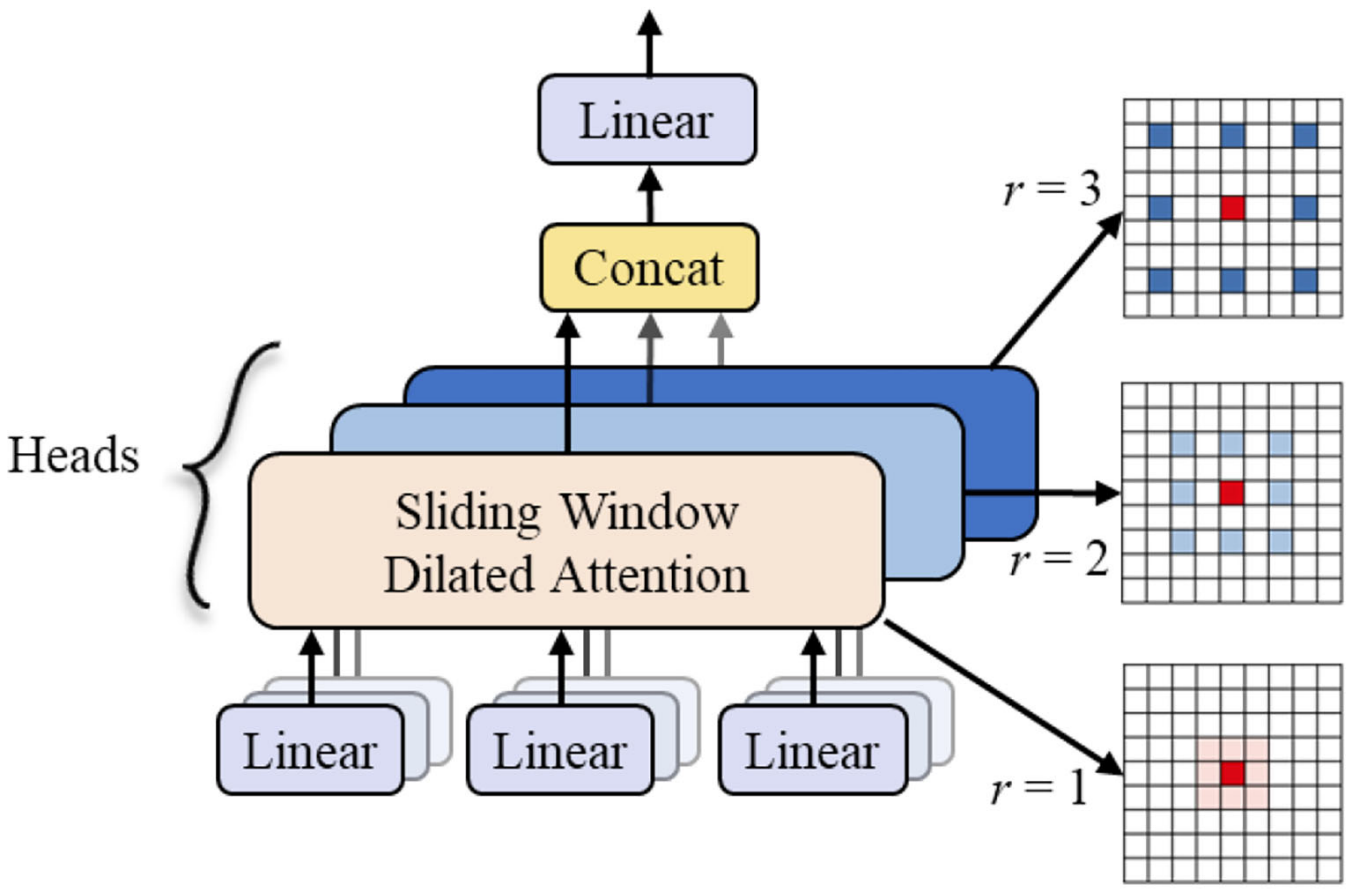
MSDA module structure.

**Figure 10** illustrates the working of the Multiscale Expanded Attention (MSDA) mechanism in the C2PSA_MSDA module. As shown in **Figure 12**, The core objective of MSDA is to enhance the model’s ability to capture fine-grained target features and high-level contextual information in complex fruit and vegetable picking environments through the Multiscale Expanded Attention (MSDA) mechanism, which is particularly suitable for handling targets with large scale differences and variable textures.

By embedding MSDA into the C2PSA module, the model achieves more efficient multi-scale feature fusion, significantly extends the model’s receptive field, and enhances robustness to occlusions and complex background interference. This improvement is crucial for high-precision fruit and vegetable recognition and localization, as targets in picking scenes often exhibit dense clusters of growth, are partially occluded by branches and leaves, or have blurred boundaries due to uneven illumination/shadowing. The C2PSA_MSDA module enables the model to adaptively focus on key regions at different scales, significantly improving detection accuracy in complex natural environments with dense branches and leaves and overlapping fruits and robustness.

#### 2.2.4 Feature fusion optimization

**Figure 13:**
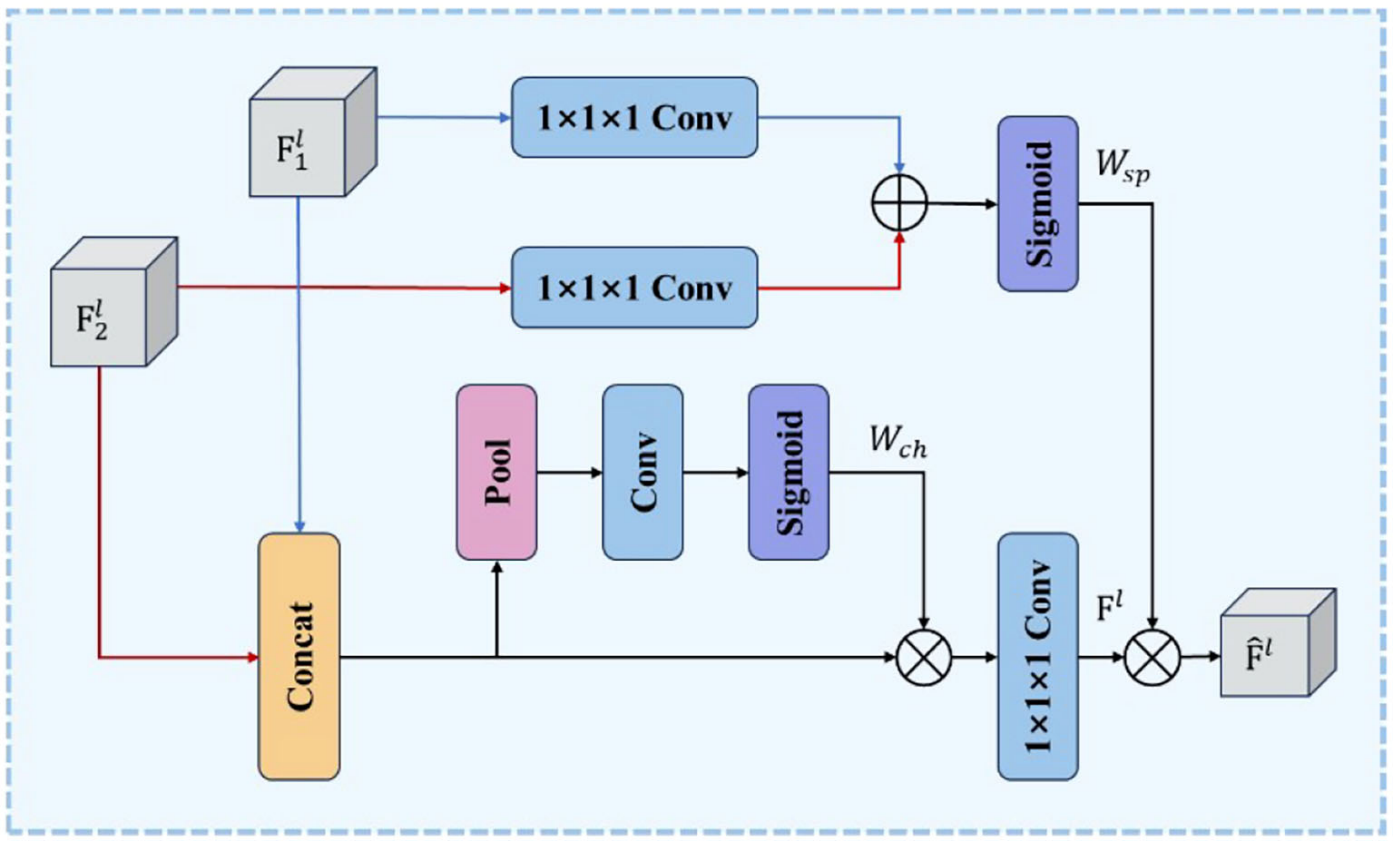
DFF module structure.

In order to optimize the multi-scale feature fusion effectiveness of the model in complex agricultural environments, this paper introduces the Dynamic Feature Fusion (DFF) module, as shown in **Figure 13**, as an alternative to the fixed-weight fusion approach of the traditional Feature Pyramid Network (FPN). The core of the DFF module lies in the use of global context information to drive the dynamic generation of feature fusion weights.

In view of the highly similar and often densely distributed or overlapping appearance of fruit for the same category in fruit and vegetable grasping scenarios, the DFF module, by virtue of its global context-awareness capability, can more effectively integrate complementary information at different scales, adaptively enhance the key feature channels for distinguishing the boundaries of densely neighboring fruit or the subtle texture differences, or strengthen the semantic information channels required for recognizing partially occluded fruit contours, so as to improve the model’s recognition accuracy of the recognition accuracy of densely distributed individuals with high similarity targets. Meanwhile, in the face of uneven illumination, changing shadows and complex background interference prevalent in the agricultural field, the global guidance mechanism of DFF enables the model to perceive the overall impact of environmental factors on the feature distribution and dynamically compensate or inhibit them in the fusion stage, which helps maintain the consistency of the target feature representations and reduces the impact of environmental interference on the detection stability.

### 2.3 Grasping point determination

**Figure 14:**
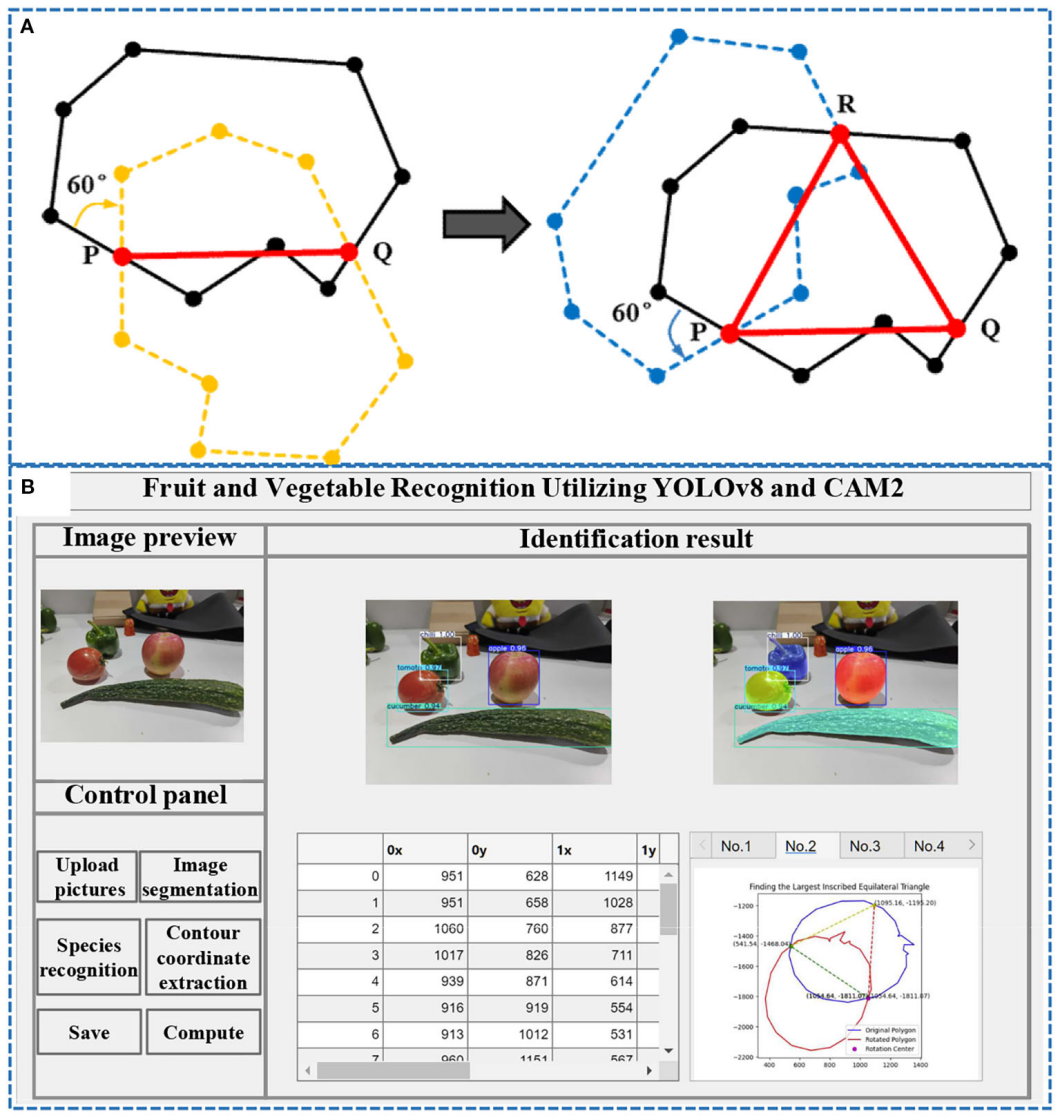
**(A)** Principle of calculating grasping points for fruit and vegetables; **(B)** Visual recognition processor.

The grasping position and posture of the soft manipulator are determined based on the geometric characteristics of the fruit and vegetables to be grasped. To enhance the efficiency and precision of fruit and vegetable screening, this study aims to improve the stability and success rate of the manipulator’s grasping actions. By integrating the FMDS-YOLOv8 algorithm with the SAM2 model, we perform contour extraction and boundary optimization for the target produce. Utilizing a “detection-first, segmentation-second” strategy, the system predicts bounding boxes and category information for all potential targets in the image, thereby achieving high-precision segmentation results. These segmentation outcomes undergo cross-validation and parameter tuning to ensure robust generalization of the model. Since the segmentation outcomes are refined through the grasping point recognition algorithm for fruit and vegetables (**Figure 14A**). This algorithm identifies optimal grasping positions for various types of fruit and vegetables, marking their centroids and contours. The visual recognition processing program developed using MATLAB software (**Figure 14B**) then calculates the grasping point positions and orientations for each actuator of the robotic manipulator, thereby obtaining the optimal grasping posture information. The underlying principles and operational steps are as follows:

The processing flow first utilizes the FMDS-YOLOv8 model for target detection and localization of fruits and vegetables in the image; subsequently, the detected bounding box is input into Segment Anything Model 2 (SAM2), which utilizes its ViT-H-based encoder-decoder architecture to generate a pixel-level segmentation mask of the target and extracts an accurate contour through the edge detection algorithm. A polygon representing the shape of the target is constructed by extracting an accurate set of contours through an edge detection algorithm. After selecting a point *P* as a reference point on this polygon, the entire polygon is rotated clockwise by 60° around point *P*. This rotation results in a rotated polygon. This rotation causes part of the rotated polygon to fall inside the original polygon and part of it to lie outside, which inevitably causes the boundaries of the two to intersect, creating at least one new intersection point *Q*. Next, the intersection point *Q* is rotated 60° counterclockwise around point *P* to give point *R* (which also lies on the boundary of the original polygon). Finally, points *P*, *Q*, and *R* form an equilateral triangle *PQR*.

The grasping point coordinates (*P*, *Q*, *R*) output by the vision system are located in a two-dimensional pixel coordinate system. These coordinates are converted into three-dimensional coordinates within the robot’s base coordinate system to guide the robotic arm’s motion. Through hand-eye calibration, the homogeneous transformation matrix *T* from the camera coordinate system to the robot’s base coordinate system is obtained. Using this matrix, points *P_cam_*, *Q_cam_*, and *R_cam_* can be transformed into the base coordinate system. Subsequently, the posture of the soft actuator is calculated. The end-effector’s posture must ensure that when the three soft fingers contact the produce, their fingertips approach perpendicular to the tangent line at points *P*, *Q*, and *R* along the fruit’s contour, thereby closing along the normal direction to complete the grasping.

### 2.4 Training setup and evaluation indicators

Computer resources are NVIDIA RTX3060 graphics card (12GB), Inter(R) Core(TM) i7 CPU processor. The deep learning framework was Pytorch 2.5.1, programming language Python 3.11.11, CUDA version 11.8, cuDNN version 8.9.7. The specific training parameters were set as follows: image input size of 640 pixels × 640 pixels, batch size of 16, multithreading set to 8, initial learning rate of 0.01, minimum learning rate of 0.0001, optimizer selection of SGD, number of training rounds set to 300, and mosaic data enhancement turned off in the last 10 rounds.

In order to accurately assess the performance of the model, this paper adopts Precision (P), Recall (R) and Mean Average Precision (mAP) as the metrics for assessing the detection accuracy of the model. In addition, the effectiveness of model lightweighting is evaluated based on FPS (Frames Per Second), inference time, and the number of model parameters (Params). Together, these metrics provide a comprehensive and detailed analysis of model performance.

### 2.5 Grasping experiments of fruit and vegetables

**Figure 15:**
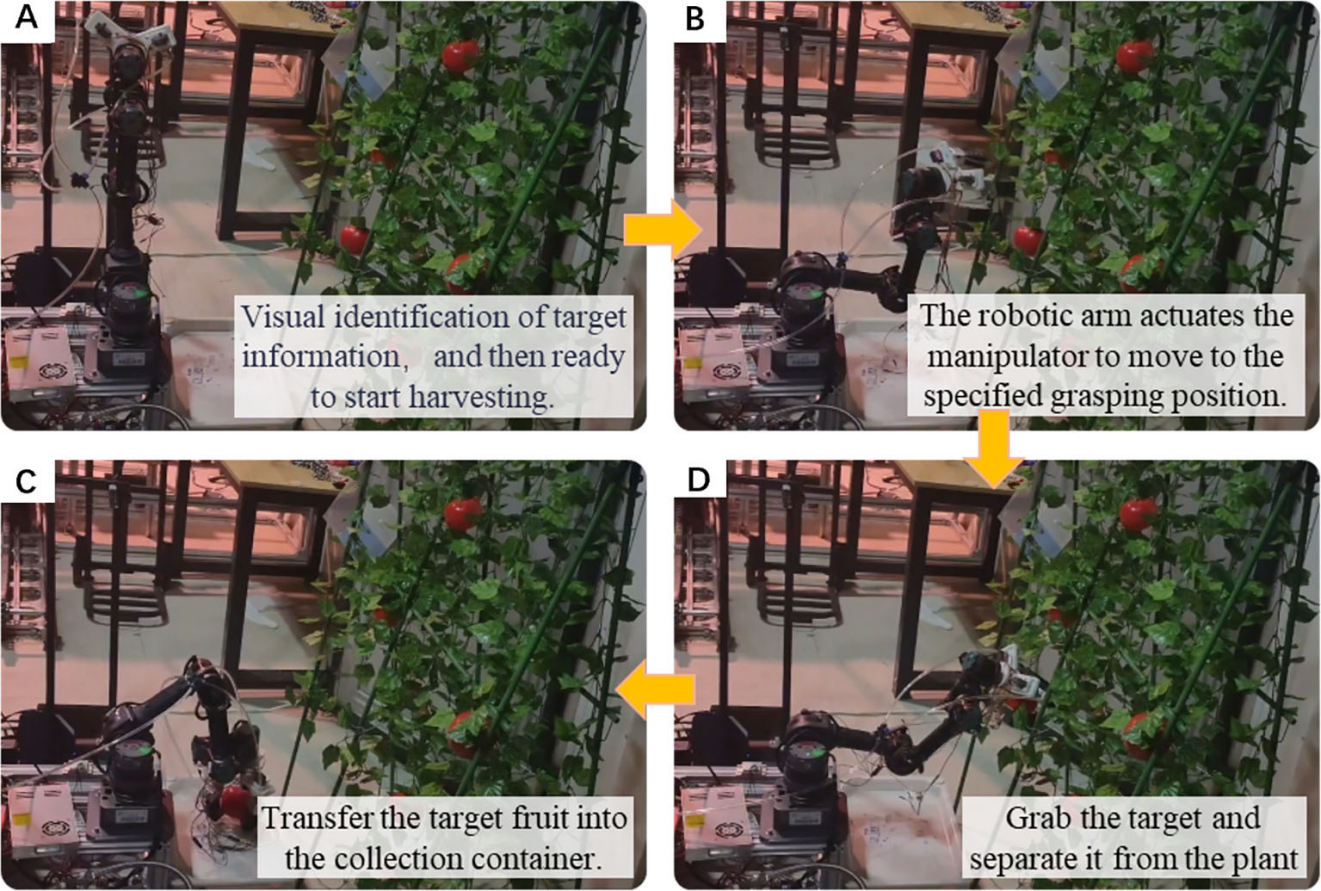
The process of soft manipulator systems for harvesting fruit and vegetables.

To evaluate the functionality of the soft MGS and the feasibility of harvesting fruit and vegetables, this study presents preliminary experiments that integrate the soft MGS onto a pre-established harvesting robot platform. The platform consists of an omnidirectional mobile base, a six-axis robotic arm, a variable-structure soft manipulator, a binocular vision recognition system, a ROS-based upper computer, and an STM32-based lower computer control system. The following sections describe the overall structure and the grasping process (**Figure 15**):

In the recognition stage, the binocular vision system obtains the position, type, morphology and size information of the target fruit and vegetables in real time, and the control system calculates the optimal angle of the actuator and the maximum grasping force parameters based on the feature data. After entering the grasping preparation stage, the ROS host drives the six-axis robotic arm to position the soft robot to the target coordinates, while the STM32 microcontroller dynamically adjusts the four-actuator configuration according to the morphology of the fruit and vegetables: for apples, oranges and other spheres, the fingers converge to the center of mass; for cucumbers and other long targets, the special grasping posture with two fingers on the same side and the third finger opposite is adopted. When the contact phase is initiated, the STM32 controls the pneumatic system to drive the soft actuator to bend with an initial air pressure of 90 kPa, and stabilized contact is achieved through the collaborative control of multiple fingers. Finally, in the picking and transportation phase, the end motor applies a tensile-torsional load to separate the fruits from the off-layer, and the soft manipulator transfers the fruits to the collection container to complete the operation.

In order to further evaluate the grasping performance of the soft manipulator adapted to the variable structure of objects of different sizes, we have conducted the grasping experiments of fruit and vegetables. In this study, we considered several representative fruit and vegetables based on factors such as size, shape, and hardness, all of which have economic value and may affect the grasping performance of the soft manipulator. Specifically, we selected cucumbers (elongated and rough surface), apples (smooth and hard surface), tomatoes (smooth and easily damaged surface), peppers and oranges (relatively soft texture) for the grasping experiments. Five samples of each fruit and vegetable were selected for the grasping experiment.

Fifty fresh samples without significant defects were prepared for each type of fruit and vegetable, totaling 250 samples to ensure statistical validity of the experiment. To prevent cumulative damage from multiple grips introducing errors into the results, each independent sample was used only for one set of specific pressure conditions during the grasping test, and the number of grips per sample was strictly limited to three. The experiment employed a controlled variable method. For each fruit or vegetable type, its 50 samples were randomly divided into 5 groups (n=10 per group), corresponding to 5 distinct grasping drive pressure levels (70 kPa, 80 kPa, 90 kPa, 100 kPa, and 110 kPa). Sample size, quantities and the number of experimental runs is shown in **Table 2**.

**Table 2:** Comparison of the accuracy of each model.

Grabbing target	Weight (g)	Mean Height (mm)	Mean Diameter (mm)	Quantity	Number of captures at each pressure	Total number of experiments
Apple	218.51 ± 29.38	77.88	79.14	50	3	150
Orange	124.26 ± 13.28	59.81	69.94	50	3	150
Tomato	139.65 ± 18.45	66.63	69.14	50	3	150
Chilli	124.15 ± 8.58	74.12	74.46	50	3	150
Cucumber	209.59 ± 29.38	298.3	38.22	50	3	150

### 2.6 Grasping damage experiment of fruit and vegetables

The experiment was carried out in September 2024 in a photoplant factory of Northeast Agricultural University, Harbin, China. Ambient temperature was 24~26°C. In the grasping experiment, we observed that once the air pressure reaches a certain threshold, further increases in air pressure have minimal impact on the success rate of grasping fruit and vegetables. However, as the driving air pressure continues to rise, it can cause damage to the skin of fruit and vegetables, particularly those that are tender and more susceptible to injury. This damage manifests as surface indentations and also reduces the operational lifespan of the soft actuator.

Due to the firm texture of apples and cucumbers, the hollow internal structure of bell peppers, and the tendency of smaller oranges to slide upward when grasping force increases. In this study, these fruit and vegetables sustained minimal damage when handled by the flexible robotic arm. Therefore, we conducted grasping damage tests exclusively on tomato fruits. However, it must be noted that for fruit and vegetables other than tomatoes, the precise, microscopic, or long-term storage-related damage observed here is qualitative in nature. Quantifying such damage requires future rigorous experimental designs.

Prior to conducting the grasping damage test, the target fruit was meticulously examined to ensure that the selected sample was intact and exhibited no signs of pre-existing damage. In this study, 51 tomatoes were selected as test subjects, comprising 48 tomatoes for the grasping test and 3 tomatoes for the control group. The sizes of the selected tomatoes were controlled within a range of 60 to 90 mm. Each tomato was held for 5 s before being released, after which damage was assessed. The grasping success rate was defined to evaluate the performance of the end effector. The damage process in tomatoes typically involves two stages: (1) mechanical damage to the fruit cell membrane and cell wall, which leads to the release of cell wall-modifying enzymes; and (2) contact between the enzyme and substrate, promoting degradation and resulting in softening and browning in the affected areas. An immediate inspection was conducted post-grasping to check for surface damage. If no obvious damage was detected, all tomatoes were stored in a controlled temperature environment for 7 days to observe potential internal damage. This study indirectly assessed internal damage through the browning rate in 72 h and shelf life of the fruit, while using the direct damage rate as an indicator of non-destructive grasping. Observations of browning rate within 72 hours are mainly for fruit and vegetable species that are susceptible to oxidative browning (e.g., tomatoes and apples), but browning may be significantly delayed for some fruit and vegetables with low PPO activity. In this study, the index of the direct damage is whether there is indentation on the fruit surface, if there is indentation, it means that some cells inside the fruit have broken (Zhang, 2021). The indicators are defined as follows:


(5) $$s=\frac{n}{m}\times 100\%$$

(6) $$D_{d}=\frac{p}{m}\times 100\%$$

(7) $$B_{s}=\frac{q}{n}\times 100\%$$

Where *s* is the grasping success rate, % as shown in Equation 5. In this study, if the target fruit does not fall during the grasping process, the grasping is considered successful; *n* is the number of successful grasping; *m* is the total number of experimental samples; *D*_d_ is the direct damage rate, %, as shown in Equation 6 and the direct damage primarily encompasses mechanical injury inflicted by the end-effector on the tomatoes, as well as damage resulting from the tomatoes falling during the grasping process; *p* is the direct damage quantity; *B*_s_ is the browning rate within 72 h, % as shown in Equation 7. Following the completion of the experiment, the tomatoes were stored for a period of 72 hours to visually assess the development of browning. Observable brown spots emerged and the affected areas exhibited softening, thereby confirming the occurrence of the browning phenomenon; *q* is the number of browned tomatoes.

## 3 Results and discussion

### 3.1 Comparison of detection models

In order to comprehensively evaluate the applicability of FMDS-YOLOv8n in fruit and vegetable grasping scenarios, this study compares and analyzes it with mainstream lightweight detection models under unified experimental conditions.

**Table 3** shows that FMDS-YOLOv8n has a combined advantage in key performance metrics. The model has a precision rate of 94.1% and a recall rate of 91.0%, which are 3.4% and 7.1% higher than the base architecture YOLOv8n, respectively. In grasping operations, the higher precision rate helps to reduce the probability of false picking of immature fruits or background objects, and the improved recall rate directly reduces the omission of ripe fruits. Its mAP@50 reach 94.0% provides a more accurate localization basis for the grasping robotic arm. On the mAP@50–90 metric, which evaluates multi-scale adaptability, the model leads the baseline model by 2.5~4.3 percentage points with a precision of 78.4%, indicating its stability in typical orchard environments, such as differences in fruit sizes, branch and leaf shading, and changes in light. In terms of parameter count and inference speed, the FMDS-YOLOv8n model contains 3.12 million parameters, which is slightly higher than that of the baseline YOLOv8n (3.01 million). This increase suggests the incorporation of additional structural components aimed at enhancing detection capability. Despite the growth in parameter size, the model maintains a competitive inference rate of 59.5 FPS. While this is lower than YOLOv8n’s 71.8 FPS, it remains considerably higher than that of YOLOv9t, which operates at 33.2 FPS, indicating that the model remains suitable for real-time applications.

**Table 3:** Comparison of the accuracy of each model.

Number	Detection models	P/%	R/%	mAP@50/%	mAP@50-90/%	Parameters	FPS
1	YOLOv8n	90.7	83.9	93.7	75.9	3,006,623	71.8
2	YOLOv5n	92.3	83.9	91.4	74.1	2,503,919	61.8
3	YOLOv9t	95.1	81.8	91.3	75.5	1,971,759	33.2
4	YOLOv11n	91.9	84.3	90.6	74.2	2,583,127	64.6
5	FMDS- YOLOv8n	94.1	91.0	94.0	78.4	3,119,530	59.5

The results also show that the baseline model has obvious limitations. YOLOv9t has the highest precision but the lowest recall, making it easy to miss target fruit in grasping scenarios; the mAP@50 of YOLOv5n is lower than that of the iterative version of YOLOv8n, indicating that its localization precision is insufficient in the area of overlapping fruit. And the original YOLOv11n has the lowest values of mAP@50 and mAP@50-90, which can hardly satisfy the precision of the grasping operation, which is difficult to meet the accuracy and robustness requirements of grasping operations. In this study, through feature fusion enhancement and dynamic sampling strategy, FMDS-YOLOv8n realizes the synergistic enhancement of precision and recall. An increase of 7.1% in the recall rate can effectively decrease the fruit loss rate, and the improvement of mAP@50–90 enhances the adaptability to complex grasping environments, which provides an effective solution for the automated grasping system.

### 3.2 Ablation experiment

In order to validate the effectiveness of the FMDS-YOLOv8 fruit and vegetable recognition network model, we conducted a series of ablation experiments on dataset based on the YOLOv8 enhancement method proposed in this study. These experiments aim to systematically assess the contribution of each enhancement to the model performance.

The ablation experiments are essential to clarify the role of each independent module on the overall model performance. By selectively removing or modifying specific modules of the model, their effects are isolated and their relative importance assessed. This study focuses on evaluating the following three enhancements: the introduction of a feature pyramid pooling module with higher accuracy, an enhanced feature extraction module, and a more flexible feature fusion module. The specific experimental configurations are detailed in **Table 4**.

**Table 4:** Comparison of ablation test accuracy.

FocalModulation	C2PSA_MSDA	C3k2_DFF	P/%	R/%	mAP/%	mAP@50-90/%	Parameters
			90.7	83.9	93.7	75.9	3,006,623
✓			90.9	77.9	86.9	72.3	3,115,042
	✓		91.8	89.5	93.6	75.9	3,271,071
		✓	89.7	85.6	91.1	74.3	2,922,135
✓	✓		92.7	86.7	93.0	76.8	3,189,650
✓		✓	90.3	90.2	92.7	74.3	3,030,554
	✓	✓	92.7	90.8	94.3	77.2	3,011,111
✓	✓	✓	94.1	91.0	94.0	78.4	3,119,530

The experimental results evaluate the performance contributions of three innovative modules, FocalModulation, C2PSA_MSDA multiscale domain attention and C3k2_DFF deep feature fusion. The experimental results show that the eighth-row configuration, i.e., the model that simultaneously integrates FocalModulation, C2PSA_MSDA and C3k2_DFF, exhibits the best overall performance. The model reaches its maximum value with 93.3% precision and 91.0% recall, and its precision and recall differ by only 2.3%, which is significantly better than the other combinations. In terms of the average precision mean of the core metrics, the model reaches a global maximum of 94.0%, which is only 0.3% lower than the 94.3% of the 7th row model, but 3.9% higher than the 90.1% of the base model. Particularly worth emphasizing, it achieved a good performance of 78.4% on the mAP@50–90 index of the rigorous high-threshold detection task is a 2.5% improvement over the third-row configuration that only adds the attention module. In terms of parameter efficiency, the model achieves this excellent performance with only 3.12M parameters. Although the number of parameters increases by 3.6% from the seventh row, it is significantly lower than the 3.27M parameters of the third-row configuration enhanced with the attention module only. This balanced property of achieving peak accuracy with moderate number of parameters makes the 8th-row model an ideal architecture for accuracy-sensitive application scenarios.

The analysis of the module synergy mechanism shows that FocalModulation lays the foundation of feature expression, C2PSA_MSDA significantly improves the recall capability, and C3k2_DFF drives the final precision breakthrough. Compared to the third-row configuration without introducing deep feature fusion, the 8th row model increases the average precision mean by 0.4% while reducing the number of parameters by 4.6%. This optimal configuration will serve as the baseline architecture for subsequent experiments.

### 3.3 Results and analysis of grasping experiment

**Figure 16:**
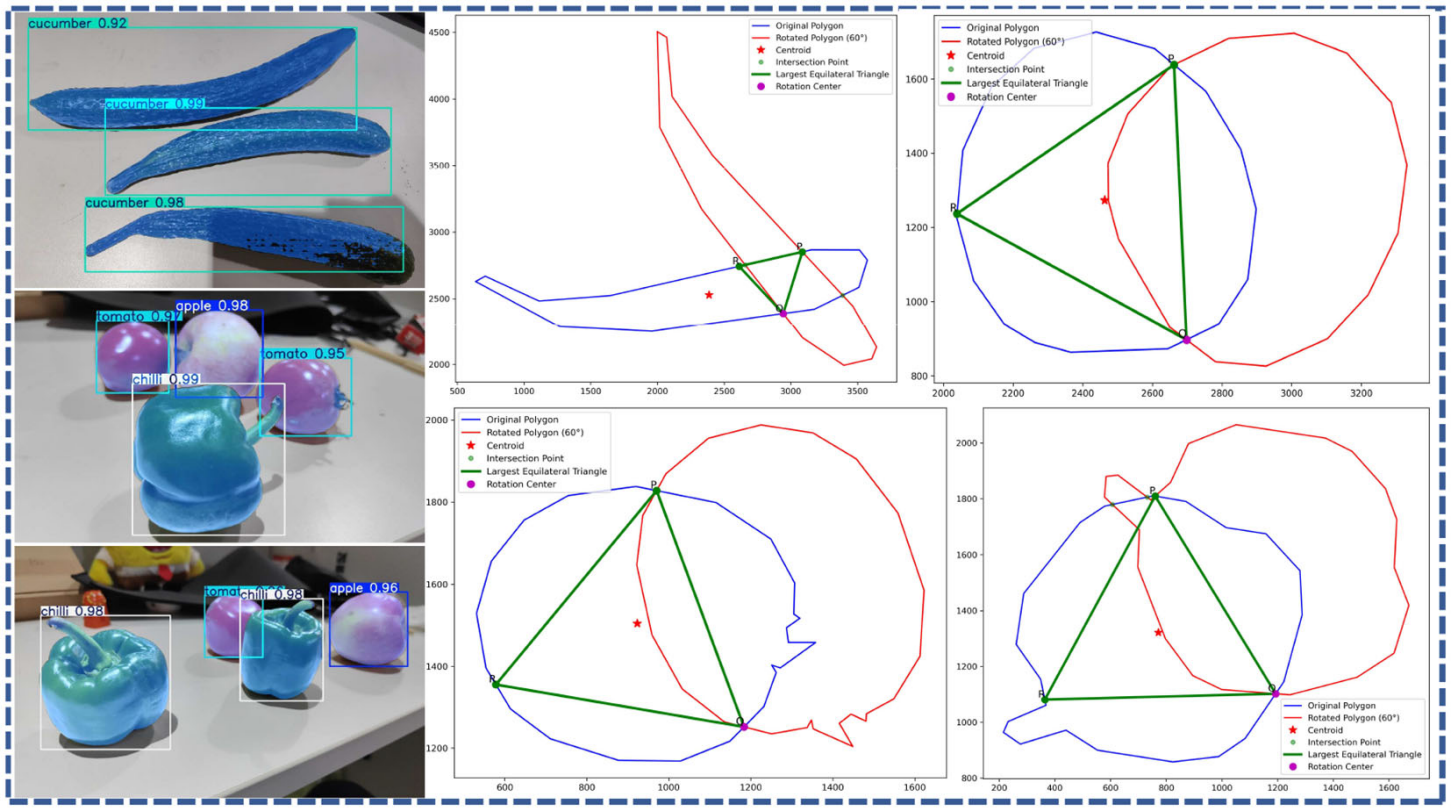
The optimal grasping point of the target fruit is determined through precise calculation.

Based on FMDS-YOLOv8 visual recognition processing algorithm, MATLAB software was used to identify and grasp a variety of fruit and vegetables. Through the operation method in Section 2.3, the fruit outline can be identified, and the radius and tangent point of the circle of the equilateral triangle inside the fruit can be determined. Consequently, parameters such as the distance between the fingers of the software manipulator and the rotational angles of the three fingers can be accurately calculated. This approach enables the effective identification of the optimal grasping point for each target fruit (**Figure 16**), thereby ensuring the stability and efficiency of the grasping operation.

**Figure 17:**
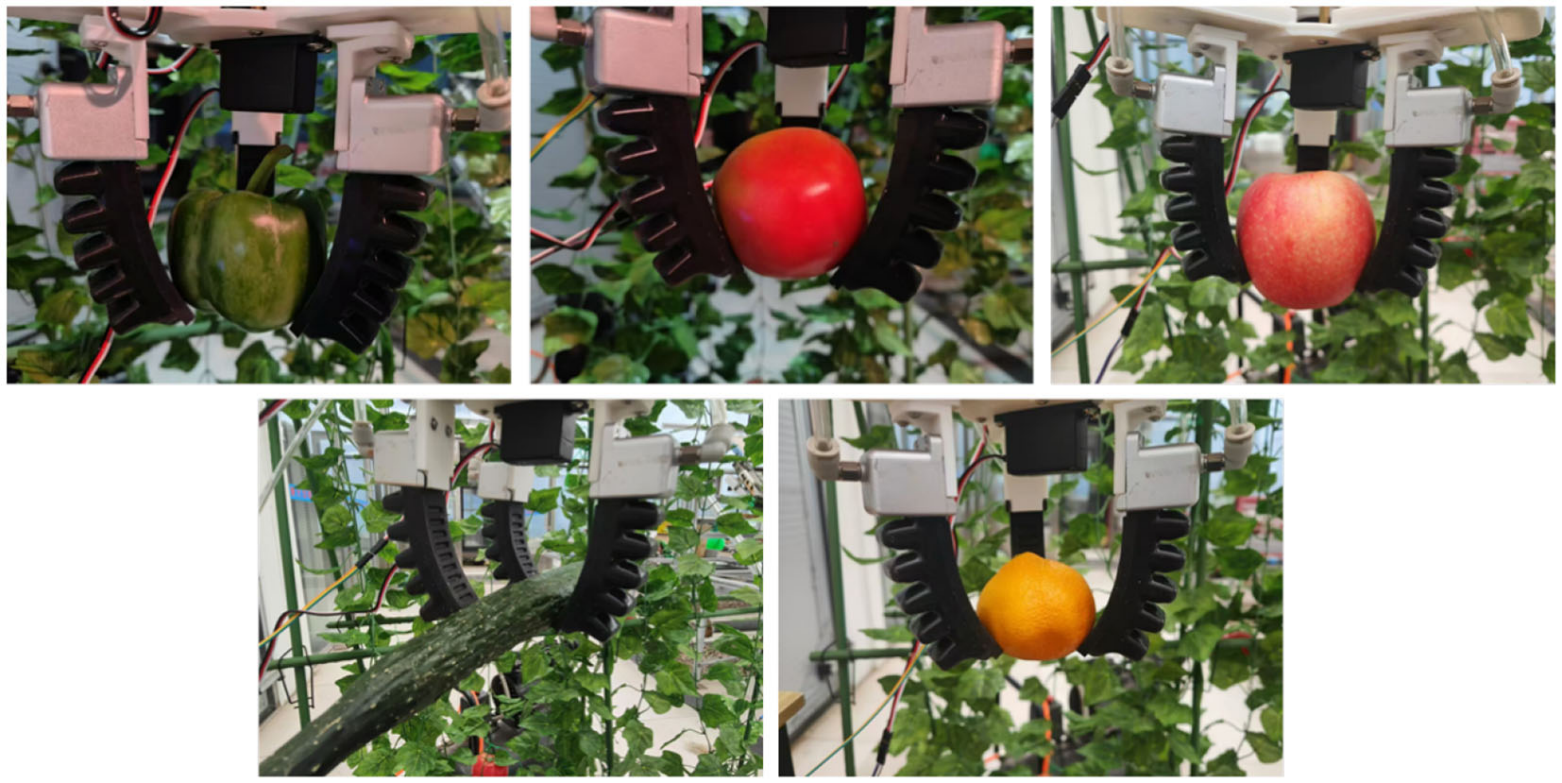
A partial and representative snapshot of the experimental outcomes.

The physical parameters and experimental results corresponding to the captured objects are shown in **Table 5**, and some representative captured results are shown in **Figure 17**.

**Table 5:** Grasping target characteristics.

Grasping target	Grasping success rate (%)				
	70 kPa	80 kPa	90 kPa	100 kPa	110 kPa
Apple	0	86.0%	100%	100%	100%
Orange	100%	100%	100%	100%	100%
Tomato	0	88.0%	100%	100%	100%
Chilli	100%	100%	100%	100%	100%
Cucumber	0	70%	73.6%	84.7%	90%

**Table 5** exhibits excellent adaptability when grasping fruit and vegetables with varying characteristics. For instance, lightweight fruit such as oranges and bell peppers can be stably gripped using only 70 kPa of driving pressure. Conversely, for smooth-surfaced, heavier items like apples and tomatoes, the driving pressure must be appropriately increased to provide sufficient grasping force and prevent slippage. Specifically, when handling cucumbers—which are heavy yet small in diameter—the grasping success rate at 90 kPa pressure was 70%. When pressure was increased to 110 kPa, the success rate significantly improved to 90%. This demonstrates that the grasping point locations identified by the vision system closely coordinate with the gripper’s grasping parameters. By dynamically adjusting the drive pressure, reliable grasping of diverse fruit and vegetables can be achieved.

Analyzing the reasons, in addition to the intrinsic attributes of the fruit such as its weight and shape, the instability in grasping operations is also attributed to the variable structure algorithm designed in this study. The potential misalignment of the center of mass for elongated fruits may lead to unstable grasping and consequently increase the risk of fruit drop. Therefore, future research efforts should focus on optimizing visual recognition and processing algorithms or enhancing the driving air pressure to ensure stable manipulation of long fruit and vegetables.

The experimental results presented above demonstrate that the soft manipulator for fruit and vegetables harvesting designed in this study exhibits excellent flexibility and adaptability. By appropriately adjusting the driving air pressure and steering gear angle, the manipulator can effectively capture fruit and vegetables of various shapes and sizes.

### 3.4 Results and analysis of grasping damage experiment

The results of grasping damage experiment are shown in **Table 6**. It can be calculated from the experimental data that the overall grasping success rate is 96%, while the direct grasping damage rate stands at 4.17%. The majority of failed grasping occurred with tomatoes that have a larger fruit diameter. This is primarily attributed to the greater weight of these larger tomatoes, which, in conjunction with insufficient grasping force from the manipulator under low driving pressure, leads to reduced clamping stability and consequently results in instances of tomato drop.

**Table 6:** Statistics of grasping damage results of tomato.

Driving air pressure	Number	Success rate of grasping (%)	Direct damage rate of clamping (%)	Browning rate in 72h (%)	Shelf life (Day)
Control group	3	–	–	–	6-7
80 kPa	16	88.0%	0	0	6-7
100 kPa	16	100%	0	0	4-6
120 kPa	16	100%	12.5%	18.75%	3-5
Average	–	96.0%	4.17%	6.25%	–

Among the 48 tomatoes in the grasping damage experiment, the browning rate at 72 hours was 6.25%. Browning or softening at 72 hours was significantly influenced by both the magnitude of the grasping force and the maturity level of the tomatoes. This study focused solely on ripe tomatoes for the grasping experiment, without investigating the effects of varying degrees of ripeness. Therefore, based on the results of the grasping success rate, direct damage rate, and browning rate, it is evident that the variable structure soft manipulator developed in this study exhibits an effective non-destructive clamping performance. In addition to fruit maturity and driving air pressure, the fruit damage rate is also influenced by factors such as the width of the mechanical gripper and the grasping contact area. For example, an insufficient finger contact area may lead to excessive localized pressure, potentially causing fruit damage. Therefore, subsequent research will focus on optimizing these structural parameters of the robotic manipulator.

The performance comparison between the variable structure soft manipulator designed in this study and the fruit and vegetable grasping device studied by previous scholars is shown in **Table 7**. Its main advantage is that it achieves multi-target compatibility grasping through variable structure (success rate is 96.0%), which is comparable to the performance of specialized rigid manipulators [e.g., tomato picking 96.03% (Chen et al., 2021)] and single soft structure [e.g., citrus grasping 96.67% (Li et al., 2024)], and it also supports diversified targets, such as spherical and irregular shapes, which significantly extends the application scenarios. The disadvantage is reflected in the fact that the grasping elapsed time (6.36 s) is 21-80% longer than the comparative study, which mainly stems from the computation and execution delays of the structure transformation, reflecting the inherent contradiction between efficiency and generalizability in adaptive grasping. Notably, the current test data are all based on laboratory environments, while the field success rates of similar studies in the table generally decrease by 5-13% (Tang, 2018; Feng et al., 2018; Xiao, 2022), highlighting the necessity of unstructured scenario validation. Compared with the prior art, this study reduces the driving complexity through the 3-finger optimized design comparing with the 4-finger structure (Ye et al., 2024) while maintaining a high success rate, and the comprehensive performance can be further improved through algorithm optimization and cross-scenario testing in the future.

**Table 7:** The main grasping performance and characteristics of the grasping manipulators.

End-effector structural form	Number of fingers	Success rate of grasping	Time-intensive	Grasping target	Test scenario	Ref. number
Rigid Manipulator	2	76.3%	5.588 s	Tomatoes	Field experiment	(Tang, 2018)
Rigid Manipulator	2	≥80%	9.6 s	Globular fruits	Laboratory	(Li et al., 2023)
Rigid Manipulator	3	96.03%	5 s	Tomatoes	Field experiment	(Chen et al., 2021)
Pneumatic soft manipulator	3	96.67%	3.54 s	Citrus	Laboratory	(Li et al., 2024)
Fin-shaped soft manipulator	3	96%	4.63 s	Citrus	Laboratory	(Xiao, 2022)
		86%			Field experiment	
Soft sleeve	–	83.9%	5.588 s	Tomatoes	Field experiment	(Brown and Sukkarieh, 2021)
Pneumatic soft manipulator	4	85%	5.34 s	Cauliflower	Laboratory	(Ye et al., 2024)
Variable structure soft manipulator	3	96.0%	6.36 s	Different shaped fruits and vegetables	Laboratory	This manuscript

Furthermore, the variable-structure soft manipulator in this study primarily targets the grasping of irregular and fragile fruits and vegetables. Consequently, during the development of the visual recognition algorithm and design of the robotic hand structure, emphasis was placed on enhancing the adaptability and robustness of the grasping system for agricultural robots. However, factors such as occlusion of fruit and vegetables and variations in lighting conditions in real-world picking environments were not fully considered during the recognition and picking processes. Therefore, future research should focus on optimizing the visual recognition algorithms and grasping control strategies to better address these challenges and meet the practical requirements of harvesting a wider variety of fruit and vegetables.

## 4 Conclusions

In this study, we designed and fabricated a variable-structure soft manipulator. This innovation optimizes the grasping strategy for irregularly shaped and delicate fruit and vegetables by actively adapting to varying structural requirements, thereby addressing the current limitations in adaptability observed in existing harvesting manipulators. To evaluate the adaptability and performance of the soft manipulator, we developed a variable structure grasping strategy based on the FMDS-YOLOv8 visual recognition algorithm. We conducted the grasping experiments on five different types of fruit and vegetables. The results indicate that the designed variable-structure soft manipulator can achieve adaptive grasping of elongated and nearly spherical fruit and vegetables through angle and spacing adjustments. Additionally, damage tests on soft and delicate tomatoes confirmed the non-destructive grasping capability of the variable structure soft manipulator. Through this research, we have successfully achieved automatic recognition and grasping of various fruit and vegetables, providing an effective solution for the automation of agricultural harvesting.

However, certain limitations remain. Laboratory environments still cannot fully replicate the complex challenges encountered in field operations, such as rapidly changing light conditions, interference from rain and fog, highly dense fruit occlusion, and motion blur caused by mobile platforms. Therefore, current performance metrics primarily reflect the model’s potential under controlled conditions, with potential performance degradation in fully open environments. To address these limitations, our future work will focus on field system deployment and testing, constructing more comprehensive field datasets that include extreme scenarios, and exploring domain adaptation methods to enhance the model’s robustness in real-world environments.

## Data Availability

The original contributions presented in the study are included in the article/supplementary material. Further inquiries can be directed to the corresponding author.
